# Prediction of Stable Surfaces of Metal Oxides through the Unsaturated Coordination Index

**DOI:** 10.1021/acsomega.3c04253

**Published:** 2023-08-01

**Authors:** Shunsaku Yasumura, Takashi Kamachi, Takashi Toyao, Ken-ichi Shimizu, Yoyo Hinuma

**Affiliations:** † Institute of Industrial Science, The University of Tokyo, Komaba 4-6-1, Meguro, Tokyo 153-8505, Japan; ‡ Department of Life, Environment and Applied Chemistry, Fukuoka Institute of Technology, 3-30-1 Wajiro-Higashi, Higashi-ku, Fukuoka 811-0295, Japan; § Institute for Catalysis, Hokkaido University, N-21, W-10, Kita, Sapporo 001-0021, Hokkaido, Japan; ∥ Department of Energy and Environment, National Institute of Advanced Industrial Science and Technology (AIST), 1-8-31, Midorigaoka, Ikeda 563-8577, Osaka, Japan

## Abstract

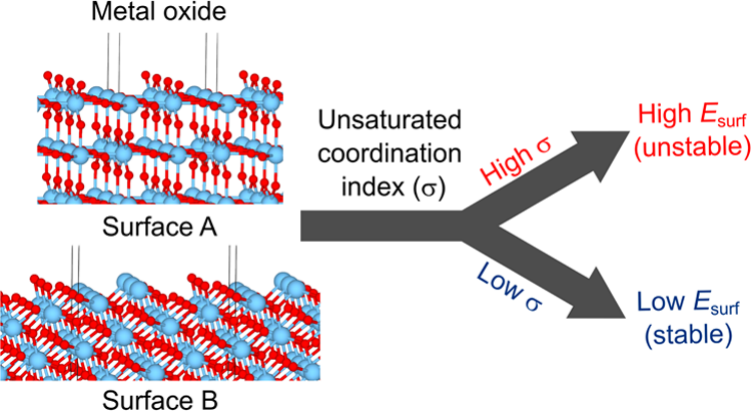

This study proposes the unsaturated coordination index, σ, as a potential descriptor of the stability of metal-oxide surfaces cleaved from bulk. The value of σ, the number of missing bonds per unit area, can be obtained very quickly using only crystallographic data, namely, the bulk geometry. The surface energies of various binary oxides, with and without atom relaxation, were calculated. Their correlations with σ had good coefficients of determination (*R*^2^) values, particularly in high-symmetry crystals. The proposed descriptor is very useful for an initial evaluation of stable metal-oxide surfaces without conducting any surface model calculations.

## 1 Introduction

Chemical reactions take place on surfaces of solid materials in heterogeneous catalysis. Much effort has been devoted to gaining atomic-scale understandings of detailed surface structures and reaction mechanisms, which is necessary for improving catalytic activity or the rational design of novel heterogeneous catalysis.^1−14^ Among solid materials, metal oxides are widely utilized in heterogeneous catalysis because of their high stabilities under experimental conditions in industrial processes, such as high temperature, pressure, and concentration of gaseous components. Slab-and-vacuum models (hereafter “slab models”) have been used to model solid surface structures within the three-dimensional periodic boundary condition to explore plausible active sites and reaction mechanisms typically based on density functional theory. A large number of cleaved terminations can be generated within a relatively small supercell size limit in low-symmetry crystals. Compared to metal surfaces,^13,14^ the surface structures of metal oxides could be very complex.^15−18^ However, most theoretical works focus on only the simplest termination, although slightly less stable surfaces are recognized to contain catalytically active sites in a limited number of individual cases. Major obstacles to the comprehensive screening of metal-oxide surfaces are the requirement of huge computational costs and the difficulty in modeling numerous accessible surfaces with different terminations. One of the authors developed algorithms to automatically generate nonpolar slab models using symmetry information.^19−21^ These efforts enabled calculations of energetic and electronic properties of slab models, including surface energies (*E*_surf_), surface defect energies (e.g., surface anion vacancy formation energies), small-molecule adsorption energies, ionic potentials and electron affinities.^20,22−32^

This study proposes the unsaturated coordination index, σ, which is the number of missing bonds, or unsaturated coordination, per unit area. This value is derived from geometrical parameters of bulk and is designed as a descriptor of *E*_surf_. The validity of σ was verified using 12 terminations, including reconstructed surfaces, of rocksalt structure MgO and CaO, six terminations of anti-fluorite structure Li_2_O, Na_2_O, and K_2_O, 16 terminations of rutile structure TiO_2_, SnO_2_, and GeO, 18 terminations of anatase TiO_2_, 67 terminations of isostructural θ-Al_2_O_3_ and β-Ga_2_O_3_, and 68 terminations of the monoclinic (m-) baddeleyite structure ZrO_2_. Good correlations between σ and *E*_surf_ were found when atom positions were fixed to cleaved bulk, and the correlations were reasonable when atom positions were relaxed. The accessible surfaces of rutile and anatase structured TiO_2_, SnO_2_, GeO_2_, and m-ZrO_2_ that do not spontaneously decompose into macroscopic facets of terminations with different orientations are also reported here.

## 2 Computational Details

### 2.1 *E*_surf_ Calculations

First-principles calculations were conducted using the projector augmented-wave method^33^ as implemented in the VASP code.^34,35^ The plane-wave basis set cutoff was 400 eV. As in our previous studies,^26,29^ the Perdew–Burke–Ernzerhof functional tuned for solids (PBEsol)^36^ within the generalized gradient approximation (GGA) was used because it provides reasonable bulk energetics and crystal structures, for instance, compared to the standard PBE-GGA functional^37^ as shown in a previous systematic study of polymorphs of groups I–VI binary oxides.^38^ Furthermore, the Hubbard *U* based on Dudarev’s formulation^39^ was additionally considered on 4d states of Ti and Zr. The effective *U* value of *U*–*J* (denoted as *U*_eff_) was set at 3 eV, which was used to obtain fitted elemental-phase reference energies (FERE) by Stevanović et al.^40^ The +*U* correction was applied to Ti and Zr even though Ti and Zr in TiO_2_ and ZrO_2_ have formally d^0^ electronic configurations because corrections would be necessary when treating defects where electrons could locally enter the d states. Internal coordinates and lattice parameters were relaxed in bulk calculations, and lattice parameters were fixed in slab model calculations. Spin polarization was considered in slab calculations with an initial ferromagnetic spin ordering.

Slab models under the three-dimensional periodic boundary condition were used to analyze surfaces, where slabs infinitely extending parallel to the *ab*-plane alternate with vacuum layers along the *c*-axis. The slab thickness in the models was larger than 14.5 Å and three repeat units, and the minimum vacuum thickness was 12 Å. In addition to cubic and tetragonal crystals, monoclinic β-Ga_2_O_3_, θ-Al_2_O_3_, and ZrO_2_ were considered in this study because their low symmetry and existence of inversion centers result in a large number of nonpolar surfaces within a reasonable range of the basal area of the slab model, *S*. The *E*_surf_ is defined as
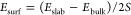
1where *E*_slab_ and *E*_bulk_ are the energy of the slab without defects and the energy of the constituents of the slab when in a perfect bulk, respectively. The coefficient of 2 accounts for both sides of the slab. Note that all used slab models are stoichiometric, and each surface (top and bottom) is symmetric.

### 2.2 Definition of the Unsaturated Coordination Index (σ)

The unsaturated coordination index, σ, is defined as follows. A stoichiometric slab-and-vacuum model (slab model) is assumed, and bonds form between pairs of atoms with a distance shorter than an arbitrary cutoff, *r*_cut_. The coordination number of atom *i*, CN_*i*_, is the number of bonds of atom *i*, or in other words, the number of atoms within a sphere of radius *r*_cut_ around atom *i*. The average CN of bulk, ⟨CN⟩_bulk_, is given by
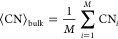
2where *M* is the number of atoms in the bulk crystal. The number of missing bonds in a slab model, ΔCN, is the number of missing CN compared to perfect bulk. When there are *N* atoms in the slab model
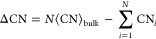
3The unsaturated coordination number is ΔCN normalized by the surface area of the slab model, which is
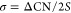
4where the factor 2 accounts for both sides of the slab.

The value of *r*_cut_ used to obtain CN_*i*_ can be any value larger than the longest “NN” bond and smaller than the shortest “second NN” bond length. Practically, the “nearest neighbor (NN)” bond length is not a fixed value but is within a certain range, except in the simplest crystals (Table 1). The calculated rutile structure TiO_2_ has two “NN” bond lengths of 1.974 and 1.998 Å. Similarly, the “second NN (2NN)” may have a range. The “2NN” in rutile TiO_2_ is a O–O bond with length 2.565 Å. There is also a 2.809 Å O–O bond, which might be considered as part of the extended “2NN” bond or a “third NN” bond, but in any case, *r*_cut_ should be smaller than 2.565 Å and bond lengths larger than this are irrelevant. Values of *r*_cut_ in this study can be any value within *R*_cut1_ and *R*_cut2_, which are arbitrary lengths larger and smaller than NN and 2NN lengths, respectively. Here, *R*_cut1_ and *R*_cut2_ were obtained with 0.1 Å intervals.

**Table 1:** Information on the Lower and Upper Boundaries of *r*_cut_, *R*_cut1_ and *R*_cut2_, Respectively^a^

crystal	*R*_cut1_ (Å)	*R*_cut2_ (Å)
MgO	2.2	2.9
CaO	2.4	3.3
Li_2_O	2.0	2.2
Na_2_O	2.4	2.7
K_2_O	2.8	3.2
rutile TiO_2_	2.0	2.5
anatase TiO_2_	2.0	2.5
SnO_2_	2.1	2.6
GeO_2_	2.0	2.4
Ga_2_O_3_	2.1	2.6
Al_2_O_3_	2.1	2.5
ZrO_2_	2.3	2.5

a Values of *R*_cut1_ and *R*_cut2_ were obtained with intervals of 0.1 Å.

A robust method to identify missing bonds for any cleaved termination was proposed by Mackenzie et al.,^41,42^ where bonds are defined as vectors and their spatial relations are evaluated against a “dividing plane”, but our procedure is more straightforward because the initial coordination of each atom does not need to be tracked and information on the direction of the bonds is not necessary.

**Figure 1:**
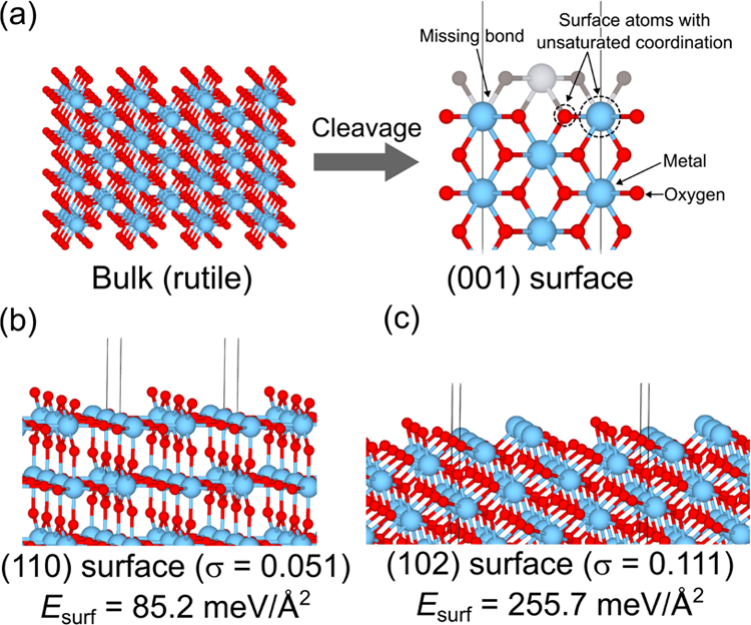
(a) Schematic showing how cleaving bulk leads to missing bonds in surface atoms, thereby resulting in unsaturated coordination. Examples of TiO_2_ terminations with (b) small and (c) large densities of missing bonds at the surface. Fixed *E*_surf_ of the terminations are also given.

Figure 1a is a schematic showing how σ is derived. When a surface structure is obtained by cleaving the bulk, the surface atoms should miss bonds from the bulk, generating surface atoms with unsaturated coordination. At this point, the surface terminations, which miss more bonds, tend to be unstable; σ reflects the density of missing bonds among the surface atoms per surface area. Figure 1b,c shows (110) and (102) surfaces of rutile structure TiO_2_ as the representatives of high and low σ terminations. A large σ indicates a greater extent of unsaturated coordination, or a higher density of missing bonds, which is expected to result in a higher *E*_surf_.

## 3 Results and Discussion

### 3.1 Details of Slab Models

#### 3.1.1 MgO and CaO

The space group of these rocksalt structure compounds is *Fm*3̅*m*. Slab models with the minimum choice of *S* less than four times that of the smallest *S*, which is for the (111) orientation, were analyzed. There is a total of six terminations of type 1 in Tasker’s definition^43^ and nonpolar type A in the definition by Hinuma et al.^19^ Additionally, the (111) surface is polar (type 3) according to Tasker but can be made nonpolar by surface reconstruction of a supercell. Three types of reconstruction patterns were considered. One is the “row” (R) pattern where one atom wide rows of one-half of surface atoms are removed. In the “zigzag” (Z) pattern, the surface atoms form a zigzag pattern when viewed from the direction normal to the surface. From another point of view, two atoms wide rows of one-half of surface atoms are removed. The “octopolar” (O) pattern has 2 × 2 ordering, where 75 and 25% of atoms in the outermost layer and second layer from the surface are removed, respectively.^44^ The outermost layer can be anions (A) or cations (C), and thus six reconstructions (combinations of A or C and R, Z, or O) were studied. The geometries of slab models and values of *E*_surf_ with fixed and relaxed atom positions are given in Tables S1 and S2, and the terminations are shown in Figure S1.

The lowest *E*_surf_ surface is (100), followed by (310), (210), and (110). As discussed afterward, the (*h*10) surfaces may be viewed as a vicinal surface of steps and terraces, with the step vector parallel to [010] and edge vector parallel to [001], on the (100) terrace plane. A smaller *h* (>0) results in a higher density of steps and, therefore, a higher *E*_surf_. The octopolar pattern has the lowest *E*_surf_ among the (111) reconstructions, as expected.^45^ The choice of the outermost element (anion or cation) does not change σ in these terminations, and the difference in *E*_surf_ was relatively small at less than 10% in the terminations considered in this study. The σ of the (111) terminations is high and does not affect the prediction of low *E*_surf_ terminations.

#### 3.1.2 Li_2_O, Na_2_O, and K_2_O

The space group of these anti-fluorite structure compounds is also *Fm*3̅*m*. Slab models with the minimum choice of *S* less than four times that of the smallest *S*, which is for the (111) orientation, were analyzed. There are three type 1 and nonpolar type A terminations, (110), (211), and (310), and three Tasker type 2 and nonpolar type B terminations, (111), (311), and (331). The geometries of slab models and values of *E*_surf_ with fixed and relaxed atom positions (fixed and relaxed *E*_surf_, respectively) are given in Tables S3–S5, and the terminations are shown in Figure S2. All missing bonds at the lowest *E*_surf_ termination, (111), are perpendicular to the surface and there are no bonds parallel to the surface. In contrast, all missing bonds at the third lowest *E*_surf_ termination, (110), are not perpendicular to the surface and there are bonds parallel to the surface. The second lowest *E*_surf_ termination, (331), has (111) termination-type steps on the (110) terrace surfaces.

#### 3.1.3 Rutile Structure TiO_2_, SnO_2_, and GeO_2_

The space group of these rutile structure compounds is *P*4_2_/*mnm*. Slab models with the minimum choice of *S* less than four times that of the smallest *S*, which is for the (001) orientation, were analyzed. 14 orientations and 16 terminations were obtained, which are all Tasker type 2 and nonpolar type B except for the (001) termination that is Tasker type 1 and nonpolar type A. The geometries of slab models and values of fixed and relaxed *E*_surf_ are given in Tables S6–S8, and the terminations are shown in Figure S3.

Surfaces subject to facet decomposition were investigated using the procedure by Hinuma et al.^46^ Surface optimization using the USPEX code^47−50^ was not performed. The terminations that did not decompose into facets are in the order of increasing relaxed *E*_surf_: TiO_2_: (110), (100), (321), (221), and (101); SnO_2_: (110), (100), (321), (221), (301), (101), and (211); and GeO_2_: (110), (100), (301), (321), (221), (211), and (101). These terminations are shown with asterisks in Tables S6–S8.

#### 3.1.4 Anatase Structure TiO_2_

The space group of anatase TiO_2_ is *I*4_1_/*amd*. Slab models with the minimum choice of *S* less than four times that of the smallest *S*, which is for the (001) orientation, were analyzed. 13 orientations and 18 terminations were obtained. There are three Tasker type 1 and nonpolar type A terminations, (100), (110), and (310), and the rest are Tasker type 2 and nonpolar type B terminations. The geometries of slab models and values of fixed and relaxed *E*_surf_ are given in Table S9, and the terminations are visualized in Figure S4. Surfaces subject to facet decomposition were investigated as in Section 3.1.3. The terminations that do not decompose into facets are in the order of increasing relaxed *E*_surf_: (101) and (001).

#### 3.1.5 β-Ga_2_O_3_ and θ-Al_2_O_3_

Compounds β-Ga_2_O_3_ and θ-Al_2_O_3_ share the same crystal structure with space group *C*2/*m*. Slab models with the minimum choice of *S* less than four times that of the smallest *S*, which is for the (100) orientation, were considered. There are 34 orientations and 67 terminations each for both Ga_2_O_3_ and Al_2_O_3_. The obtained surfaces are type 2, or nonpolar type B except for the (010) surface that was Tasker type 1 and nonpolar type A, respectively. The geometries of slab models are given in Tables S10 and S11, and the terminations are illustrated in Figures S1–S9 of ref (29).

#### 3.1.6 m-ZrO_2_

The space group of m-ZrO_2_ is **P**21/*c*. Slab models with the minimum choice of *S* less than three times that of the smallest *S*, which is for the (001) orientation, were analyzed. 28 orientations and 68 terminations were obtained, which are all Tasker type 2 and nonpolar type B. The geometries of slab models and values of fixed *E*_surf_ are given in Table S12, and the terminations are shown in Figures S5–S9.

Surfaces subject to facet decomposition were investigated according to the procedure in Hinuma et al.^46^ Slab calculations with atom positions relaxed could not be calculated for a number of slabs in Table S12. Slabs with different thicknesses were available to obtain *E*_surf_ in some of those cases, and the geometries of slabs for relaxation calculations are given in Table S13. The terminations that did not decompose into facets are in the order of increasing relaxed *E*_surf_: (111̅)A, (111)B, (211̅)A, (102)A, (110)B, (112̅)C, (10)A, (012)A, and (012̅)B (identical terminations), (001)B, (100)A, and (120)D. These surfaces are shown with asterisks in Table S12. Here, surface optimization using the USPEX code^47−50^ was conducted for the third–sixth lowest *E*_surf_ terminations, (211̅), (102), (110), and (112̅). To reduce the computational cost, the General Utility Lattice Program (GULP) code^51,52^ was used as the energy calculator in USPEX calculations together with interatomic potentials by Woodley et al.^53^ Considering the four USPEX-relaxed terminations results in facet decomposition of (102̅) to (112̅) and (11̅2̅).

### 3.2 Correlation of σ and *E*_surf_

**Figure 2:**
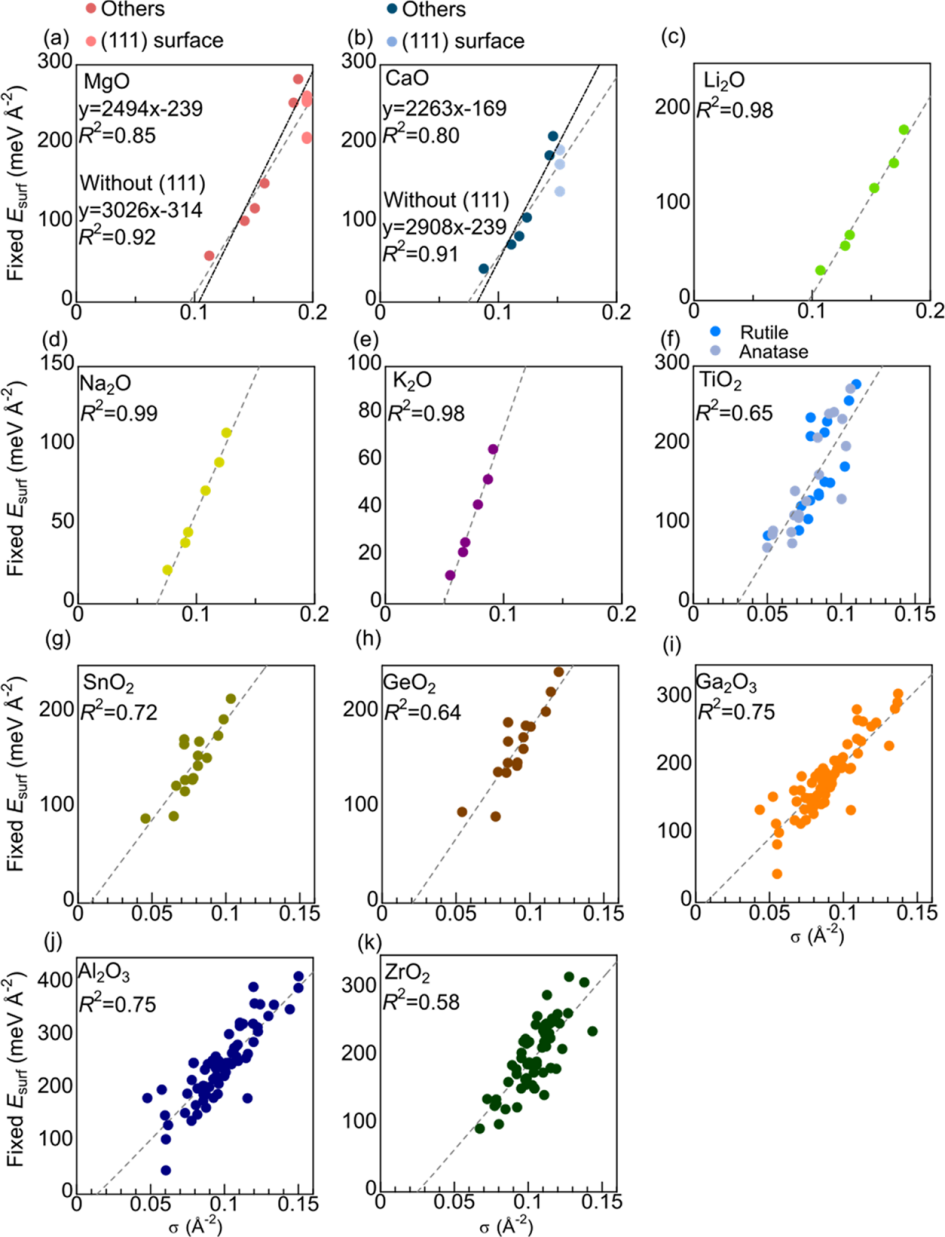
Relation between σ and *E*_surf_ when atom positions are fixed to cleaved bulks of (a) MgO, (b) CaO, (c) Li_2_O, (d) Na_2_O, (e) K_2_O, (f) TiO_2_, (g) SnO_2_, (h) GeO_2_, (i) Ga_2_O_3_, (j) Al_2_O_3_, and (k) ZrO_2_.

**Figure 3:**
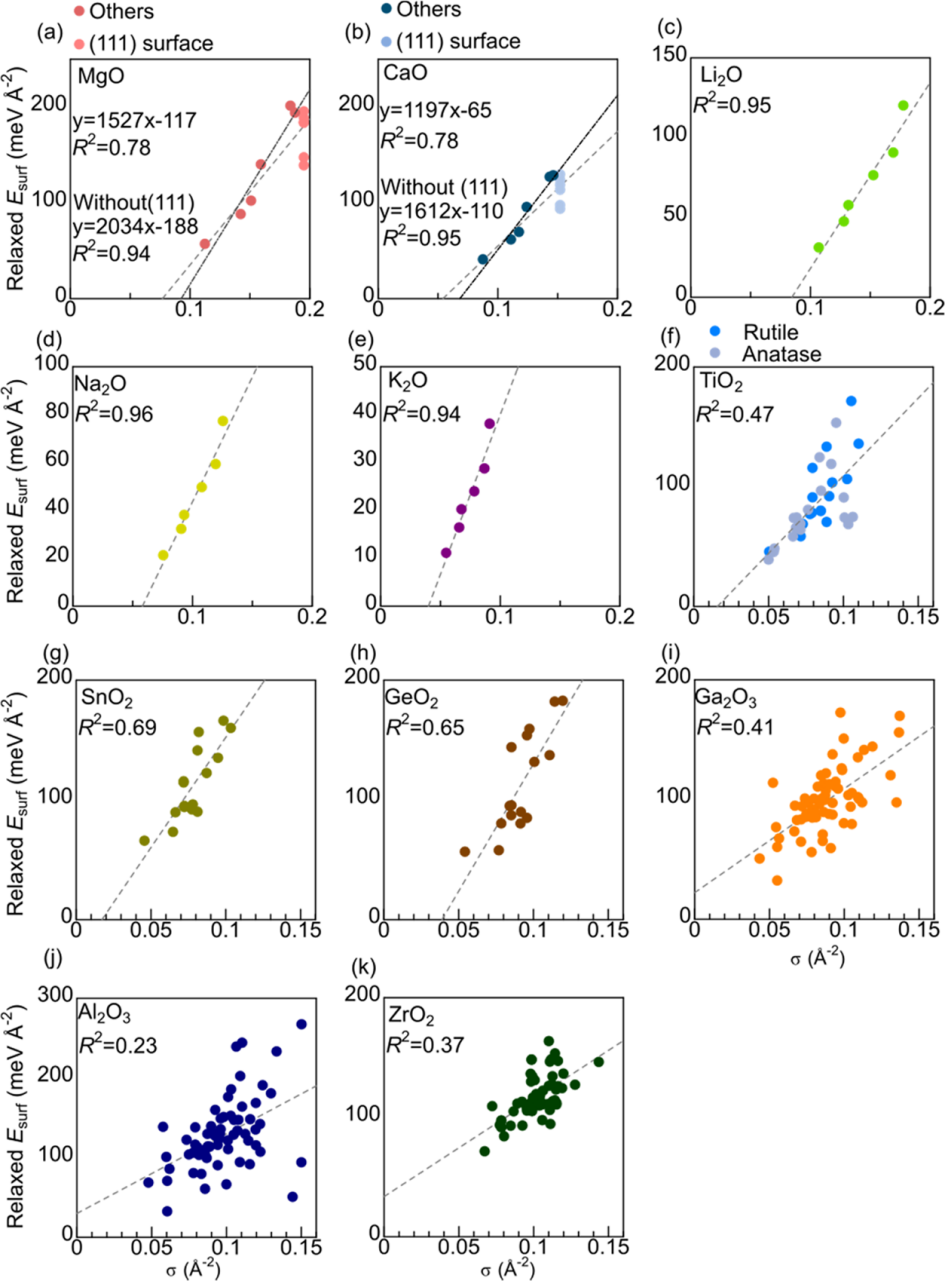
Relation between σ and *E*_surf_ when atom positions are relaxed form cleaved bulks of (a) MgO, (b) CaO, (c) Li_2_O, (d) Na_2_O, (e) K_2_O, (f) TiO_2_, (g) SnO_2_, (h) GeO_2_, (i) Ga_2_O_3_, (j) Al_2_O_3_, and (k) ZrO_2_.

Figure 2 plots the fixed *E*_surf_ against σ, which would reflect the effect of σ more than the relaxed ones. Cubic crystals are discussed first. The coefficients of determination (*R*^2^) in rocksalt structure MgO and CaO are good at 0.85 and 0.80, respectively (Figure 2a,b). The nonpolar type C (111) terminations, which appear as three distinct dots at the same σ, are below the trend of the remaining nonpolar type A terminations. The *R*^2^ for the nonpolar type A terminations only are 0.92 and 0.91 for MgO and CaO, respectively. The reconstructions for the (111) terminations cannot be distinguished using σ, but the highest *E*_surf_ termination among the six (111) terminations is 27 and 38% larger than the lowest *E*_surf_ termination in MgO and CaO, respectively. Further estimation of the *E*_surf_ requires additional descriptors from alternative approaches. The *R*^2^ in anti-fluorite structure Li_2_O, Na_2_O, and K_2_O (Figure 2c–e, respectively) is very high at 0.98 or 0.99. The order of *E*_surf_ among the six nonpolar type A or B terminations are correctly obtained in MgO, CaO, Li_2_O, Na_2_O, and K_2_O. The order is also correct for relaxed *E*_surf_ (Figure 3a–e, respectively) except for the two highest relaxed *E*_surf_ terminations in MgO. The *R*^2^ of relaxed *E*_surf_ is lower than that of the fixed *E*_surf_, but the order of relaxed *E*_surf_ can still be estimated reliably using bulk crystal structure information only.

An excellent *R*^2^ is not necessarily good news for σ. If every missing bond carries the same energy penalty, the energy penalty per unit surface area when atoms are fixed to cleaved bulk, *E*_surf_, should be proportional to the density of missing bonds per unit surface area, which is σ. In other words, *E*_surf_ = *k*σ should hold, where *k* is a crystal-dependent coefficient. This is clearly not the case in cubic crystals. For instance, the relation between *E*_surf_ and σ of MgO is *E*_surf_ = 2908σ – 239. The range of *E*_surf_ and σ lies between roughly 0 and 300 meV/Å^2^ and 0.1 and 0.2 Å^–2^, respectively, while the intercept of the regression line at σ = 0 is extremely low at −239 meV/Å^2^.

**Figure 4:**
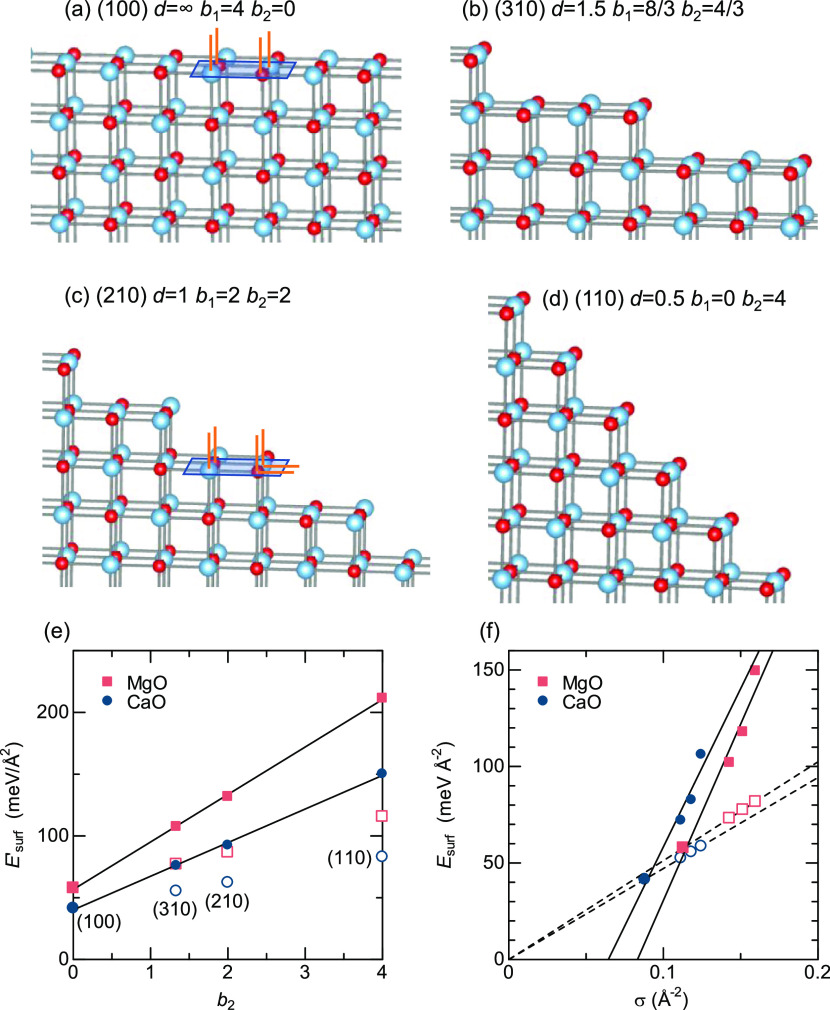
(a) (100) termination of the rocksalt structure and (b–d) (310), (210), and (110) terminations visualized as steps on the (100) terrace surface. The step interval in units of the lattice parameter *a* is *d*, and the number of atoms with one and two missing bonds per terrace area of *a*^2^ is *b*_1_ and *b*_2_, respectively, The terrace area of *a*^2^ is indicated as a blue parallelogram, and missing bonds are shown as orange lines in panels (a) and (c). (e) Surface energy per terrace surface area, *E*′_surf_, for the (100), (310), (210), and (110) terminations plotted against the number of atoms missing two bonds per terrace area of *a*^2^. (f) The corresponding surface energies per surface area, *E*′_surf_, are plotted against σ. In panels (e) and (f), filled symbols indicate actual surface energies, and the empty symbols represent hypothetical surface energies where the surface energy is proportional to the missing bond density and the energy required to break a bond is always the same as that in the (100) termination.

The *E*_surf_ of (100), (310), (210), and (110) can be described very well using a steps-on-terraces model, which is informative in understanding the possible cause of the nonzero intercept. Figure 4a shows the clean (100) surface, and Figure 4b–d illustrates the (310), (210), and (110) surfaces as steps forming on the (100) terrace plane. The step interval in units of the lattice parameter *a*, *d*, the number of atoms with one and two missing bonds per terrace area of *a*^2^, *b*_1_, and *b*_2_, and surface energy per terrace area, , are shown for MgO, with atom positions fixed to cleaved bulk, in Table 2. Note that although we refer to *E*′_surf_ as the “surface energy”, it is defined as the energy penalty per terrace area upon surface formation, to be exact, not the surface energy of the terrace (100) and vicinal (310), (210), and (110) surfaces. Atoms with one missing bond are on the terraces, whereas those with two missing bonds are at the step edges, as illustrated in Figure 4a,c. The ratio of step edge atoms increases, and therefore *b*_2_ increases, as *h* in (*h*10) decreases. *E*′_surf_ vs *b*_2_ is shown for MgO and CaO in Figure 4e. There is a clear linear relationship, which is consistent with the assumption that *E*′_surf_ is determined by a sum of energy penalties in the linear form *E*′_surf_ = *E*_1_*b*_1_ + 2*E*_2_*b*_2_. Here, *E*_1_ and *E*_2_ are energies required to break a bond in atoms that end up with one and two missing bonds, respectively. Using *b*_1_ + *b*_2_ = 4 based on the number of atoms per terrace area of *a*^2^, the above equation can be rewritten as *E*′_surf_ = 4*E*_1_ + {*E*_1_ + 2(*E*_2_ – *E*_1_)}*b*_2_. The value of *E*_1_ is obtained from the intercept at *b*_2_ = 0. In a hypothetical world where *E*_2_ = *E*_1_, the surface energy per terrace area of *a*^2^ is *E*′_surf_hyp_ = 4*E*_1_ + *E*_1_*b*_2_ using *E*_1_, which is shown with empty symbols in Figure 4e. The slope for the actual surface energy, *E*_1_ + 2(*E*_2_ – *E*_1_) is substantially larger than that for the hypothetical surface energy, *E*_1_. This result suggests that breaking the second bond has a much larger energy penalty than breaking the first bond (*E*_2_ ≫ *E*_1_), which is consistent with chemical intuition. The hypothetical surface energy per surface area, , for MgO and CaO is plotted against σ in Figure 4f using empty symbols. The linear fit of empty symbols in Figure 4f passes the origin by definition. These results demonstrate that the differences in bond breaking energies between various terminations are reflected as a linear relation between *E*_surf_ and σ, suggesting that σ is an excellent descriptor of *E*_surf_ in these rocksalt structure crystals despite the fact that the original underlying assumption that the same energy is required to break any bond does not hold. A more substantial intercept means that the assumption is less valid. The steps-on-terrace analysis clarified the origin of the high *R*^2^ linear fit with a very low intercept, but such discussion can be applied only to a subset of terminations with steps in the same direction on the same terrace termination. An infinite combination of step directions and terrace orientations is necessary to describe the full set of orientations. Therefore, using additional structural information, such as the coordination of atoms at the surface, was not considered in our search for *E*_surf_ descriptors.

**Table 2:** Information Used for the Analysis of MgO in Figure 4

termination	*d*	*b*_1_	*b*_2_	*E*_surf_ (eV)	*E*′_surf_ (eV)	*E*′_surf_hyp_ (eV)	*E*_surf_hyp_ (eV)	σ (Å^–2^)
(100)	∞	4	0	57.8	57.8	57.8	57.8	0.113
(310)	1.5	8/3	4/3	102.0	107.5	77.1	73.1	0.143
(210)	1	2	2	117.9	131.8	86.7	77.5	0.151
(110)	0.5	0	4	149.5	211.4	115.6	81.7	0.160

To evaluate the performance of this new explanatory variable, we tested it for more complex oxide surfaces in addition to the cubic systems with high symmetry. In tetragonal crystals, the two lowest fixed *E*_surf_ terminations, (110) and (100), in rutile structure TiO_2_, SnO_2_, and GeO_2_ are the terminations with the smallest and second smallest σ (Figure 2f–h, respectively), and the lowest fixed *E*_surf_ termination in anatase TiO_2_, (101)A, has the smallest σ (Figure 2f). The range of points in rutile and anatase structure TiO_2_ are shown together in Figure 2f and these overlap, suggesting that the bond strengths are similar in the two polymorphs. The *R*^2^ is between 0.64 and 0.72, which is lower than the cubic crystals but still reasonable. The results using relaxed *E*_surf_ (Figure 3f–h) show similar trends but with slightly lower *R*^2^.

In addition, monoclinic crystals were also studied. The lowest fixed *E*_surf_ termination in Al_2_O_3_ and Ga_2_O_3_, (100)A, has the fourth smallest σ (Figure 2i,j, respectively). The lowest fixed *E*_surf_ termination of ZrO_2_, (111)A, has the smallest σ (Figure 2k). These statements also hold for relaxed *E*_surf_ (Figure 3i–k). The *R*^2^ for fixed *E*_surf_ is relatively high at between 0.58 and 0.75, and relaxing atoms reduces *R*^2^ to between 0.23 and 0.41. The intrinsic limitation of σ, which is how the missing bonds are distributed among the surface atoms is not considered, becomes more problematic in low-symmetry crystals. For example, the σ of (100)A and (100)B in Al_2_O_3_ and Ga_2_O_3_ are the same, but the *E*_surf_ of (100)A is roughly double of (100)B. The missing cation–O bonds are found in originally fourfold and threefold coordinated O in (100)A and (100)B, respectively, and more energy is typically required when breaking bonds from a less coordinated atom because the bonds are stronger. That being said, very low *E*_surf_ terminations tend to have low σ, which makes σ a usable descriptor.

The *E*_surf_ vs σ plots discussed above tend to correlate linearly for both fixed and relaxed *E*_surf_. Therefore, the coefficient of determination (*R*^2^) was discussed primarily as a measure of error. The descriptor σ is useful when the order of terminations sorted by *E*_surf_ is close to that sorted by σ, and thus a linear correlation is not necessary for this purpose. However, a linear correlation helps the estimation of the absolute value of *E*_surf_ for a specific surface with a certain σ when the linear trend between *E*_surf_ and σ was derived from some trial calculations. Other statistical methods, such as the (absolute) mean error and the standard deviation of the error from the linear fit, may be scientifically interesting but are not considered because practical usage is very limited.

The five terminations, in ascending order, with smallest σ, five lowest fixed *E*_surf_, and five lowest relaxed *E*_surf_ for each oxide, are summarized in Tables S14–S25. There is excellent consistency in the rocksalt and fluorite structure oxides, and the order is reproduced somewhat in rutile and anatase structure oxides. However, the prediction performance is limited in Ga_2_O_3_, Al_2_O_3_, and ZrO_2_ with monoclinic symmetry because there are more terminations with close *E*_surf_ compared to high-symmetry crystals. The lowest fixed *E*_surf_ have very low relaxed *E*_surf_ in the studied oxides, and thus once the former is correctly identified, the latter is identified too.

**Figure 5:**
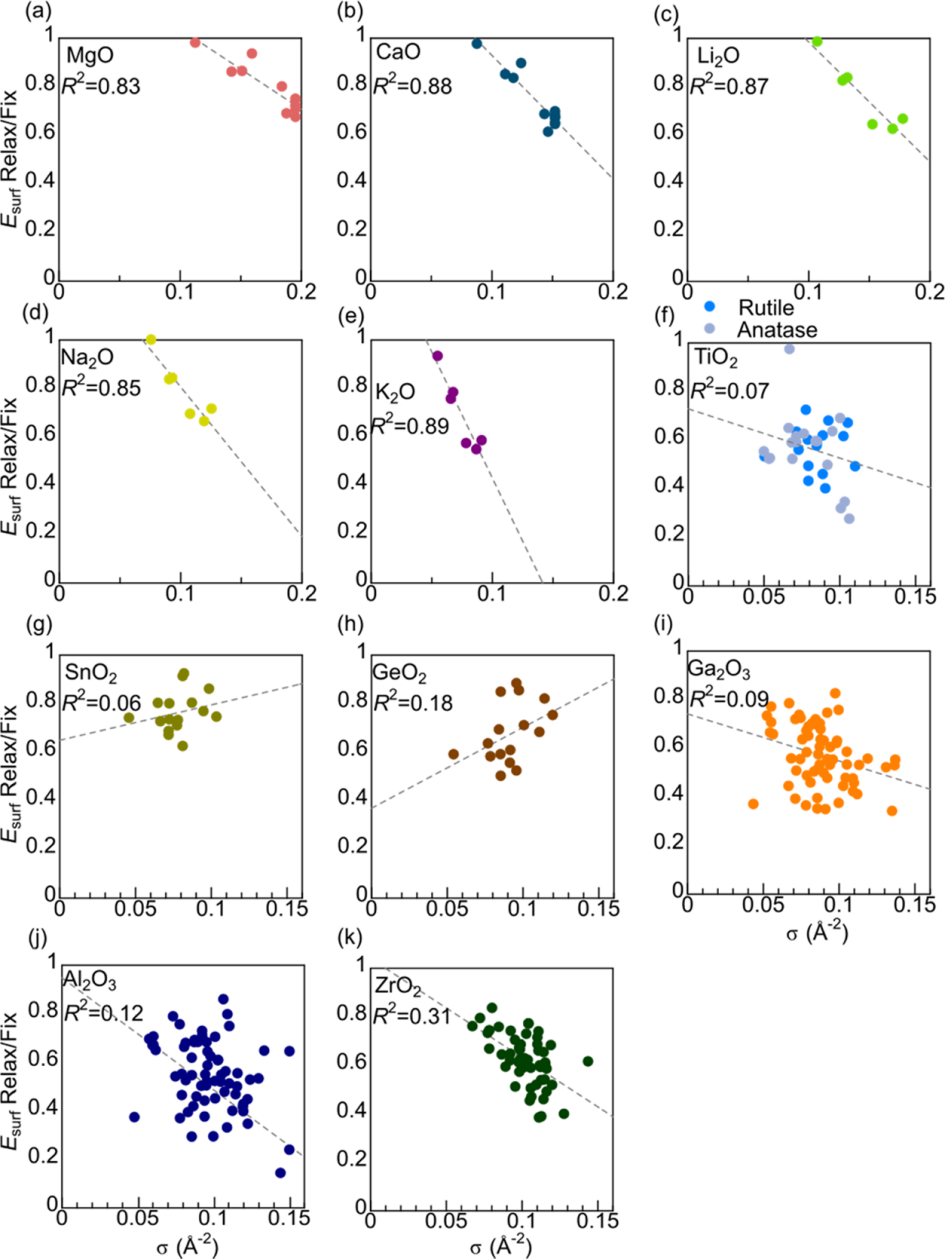
Relation between σ and the ratio of *E*_surf_ between atom positions when relaxed and fixed.

Figure 5 plots σ against the ratio of *E*_surf_ between relaxed and fixed atom positions. The cubic crystals (Figure 5a–e) have high *R*^2^ values of more than 0.8, whereas those of the others are poor at about 0.3 at most. Intuitively, a larger unsaturation of coordination, or larger σ, implies more room for relaxation, thereby reducing the ratio. This is clearly observed in the cubic crystals but not obvious in other crystals. One reason may be that the high symmetry of cubic crystals limits the variation of relaxation routes; existence of diverse relaxation opportunities would result in different extents of relaxation for surfaces with similar *E*_surf_ or σ and thereby worsening the correlation between the ratio and σ.

Finally, we discuss another limitation of σ. The values of fixed and relaxed *E*_surf_ are plotted together for all considered surfaces in Figures S10 and S11, respectively. Points for the rocksalt and anti-fluorite structures are consistently below points for the other structures, and thus the absolute value of *E*_surf_ cannot be estimated from the value of σ.

## 4 Summary

The unsaturated coordination index, σ, was defined as the number of missing bonds, or unsaturated coordination, per unit area. The validity of σ as a descriptor of *E*_surf_ was verified using 12 terminations, including reconstructed surfaces, of rocksalt structure MgO and CaO, six terminations of anti-fluorite structure Li_2_O, Na_2_O, and K_2_O, 16 terminations of rutile structure TiO_2_, SnO_2_, and GeO, 18 terminations of anatase TiO_2_, 67 terminations of isostructural θ-Al_2_O_3_ and β-Ga_2_O_3_, and 68 terminations of monoclinic baddeleyite structure ZrO_2_. Very good correlations of 0.80 or higher between fixed *E*_surf_ and σ were found for the cubic crystals, and the correlation was still good at 0.58 or higher for other crystals. The lowest *E*_surf_ termination, for both fixed and relaxed *E*_surf_, consistently had small σ. The underlying assumption is that σ is proportional to fixed *E*_surf_ when breaking of any bond carries the same energy penalty, which is not obviously true, but still σ turns out to be a reasonable descriptor of both fixed and relaxed *E*_surf_. The developed σ contributes to the computational design of solid materials, such as heterogeneous catalysts, from unexplored surfaces of metal oxides. While the user-defined parameters *R*_cut1_ and *R*_cut2_ continue to introduce a level of arbitrariness in the missing bonds, the development of a simplified descriptor is necessary to describe crystal structures effectively. The proposed descriptor σ is intrinsically more effective when the nature of bonds is not diverse. Alternate descriptors need to be designed to predict low *E*_surf_ with further confidence, especially in crystals with diverse types of bonds.

## Supporting Information Available

Information on the used slab models; structure of the considered surface; and plots of fixed and relaxed *E*_surf_ for all considered surfaces against σ (PDF)
