# Supplementary material for: Prediction of Stable Surfaces of Metal Oxides through the Unsaturated Coordination Index
Source: ACS Omega. 2023 Aug 1;8(32):29779–88. doi: 10.1021/acsomega.3c04253 (PMC10433516; doi:10.1021/acsomega.3c04253)
Supplement: Supplementary file 1 — ao3c04253_si_001.pdf [file ao3c04253_si_001.pdf]

Supplementary Material for:

Prediction of stable surfaces of metal oxides through the unsaturated coordination index

Shunsaku Yasumura[a], Takashi Kamachi[b], Takashi Toyao[c], Ken-ichi Shimizu[c], and Yoyo Hinuma[d*]

[a] Institute of Industrial Science, The University of Tokyo, Komaba 4-6-1, Meguro, Tokyo 153-8505, Japan

[b] Department of Life, Environment and Applied Chemistry, Fukuoka Institute of Technology, 3-30-1Wajiro-Higashi, Higashi-ku, Fukuoka 811-0295, Japan

[c] Institute for Catalysis, Hokkaido University, N-21, W-10, Kita, Sapporo, Hokkaido 001-0021, Japan

[d] Department of Energy and Environment, National Institute of Advanced Industrial Science and Technology (AIST), 1-8-31, Midorigaoka, Ikeda, Osaka 563-8577, Japan

Corresponding author

*Yoyo Hinuma (y.hinuma@aist.go.jp)

Supplementary Information. Detailed data of slab models.

Table S1. Geometry of MgO slabs.

Termination	a (Å)	b (Å)	γ (°)	Area A (Å^2)	Height (Å)	Occupancy	#atoms/ cell	E_{surf} (meV/ Å^2)	
								Fix	Relax
(100)	2.98	2.98	90.0	8.9	29.5	0.571	16	57.8	56.6
(110)	2.98	4.21	90.0	12.5	28.3	0.579	22	149.5	139.7
(111)AR	5.16	2.98	90.0	15.3	31.6	0.615	32	260.4	195.0
(111)AZ	5.16	5.95	90.0	30.7	31.6	0.615	64	255.5	183.7
(111)AO	5.95	5.95	120.0	30.7	31.6	0.577	60	205.7	138.7
(111)CR	5.16	2.98	90.0	15.3	31.6	0.615	32	252.2	188.2
(111)CZ	5.16	5.95	90.0	30.7	31.6	0.615	64	254.5	183.1
(111)CO	5.95	5.95	120.0	30.7	31.6	0.577	60	208.2	147.0
(210)	4.21	5.16	65.9	19.8	27.3	0.586	34	117.9	101.7
(211)	2.98	7.29	90.0	21.7	27.5	0.563	36	251.4	200.9
(221)	2.98	8.93	90.0	26.6	27.4	0.564	44	281.2	193.6
(310)	4.21	6.66	90.0	28.0	28.0	0.571	48	102.0	87.7

Table S2. Geometry of CaO slabs.

Termination	a (Å)	b (Å)	γ (°)	Area A (Å^2)	Height (Å)	Occupancy	#atoms/ cell	E_{surf} (meV/ Å^2)	
								Fix	Relax
(100)	3.37	3.37	90.0	11.4	28.6	0.583	14	41.5	40.4
(110)	3.37	4.77	90.0	16.1	28.6	0.588	20	106.2	95.1
(111)AR	5.84	3.37	90.0	19.7	33.0	0.583	28	192.1	129.2
(111)AZ	5.84	6.74	90.0	39.3	33.0	0.583	56	191.4	123.3
(111)AO	6.74	6.74	120.0	39.3	33.0	0.542	52	139.3	97.1
(111)CR	5.84	3.37	90.0	19.7	33.0	0.583	28	173.2	118.3
(111)CZ	5.84	6.74	90.0	39.3	33.0	0.583	56	173.8	113.2
(111)CO	6.74	6.74	120.0	39.3	33.0	0.542	52	139.0	93.2
(210)	4.77	5.84	65.9	25.4	27.7	0.577	30	82.7	69.1
(211)	3.37	8.25	90.0	27.8	27.2	0.571	32	184.7	126.7
(221)	3.37	10.11	90.0	34.1	27.8	0.571	40	209.2	128.2
(310)	4.77	7.54	90.0	35.9	27.9	0.568	42	72.1	61.3

Table S3. Geometry of Li_2O slabs.

Termination	a (Å)	b (Å)	γ (°)	Area A (Å^2)	Height (Å)	Occupancy	#atoms/ cell	E_{surf} (meV/ Å^2) Fix	Relax
(110)	3.25	4.60	90.0	14.9	27.6	0.588	30	68.6	57.4
(111)	3.25	3.25	60.0	9.1	31.8	0.583	21	31.3	30.8
(211)	3.25	8.60	67.8	25.9	27.2	0.586	51	118.1	76.1
(310)	4.60	7.27	90.0	33.4	27.6	0.579	66	179.5	119.8
(311)	3.25	5.63	73.2	17.5	29.1	0.571	36	144.3	90.3
(331)	3.25	7.27	77.1	23.0	30.6	0.621	54	57.2	47.2

Table S4. Geometry of Na_2O slabs.

Termination	a (Å)	b (Å)	γ (°)	Area A (Å^2)	Height (Å)	Occupancy	#atoms/ cell	E_{surf} (meV/ Å^2) Fix	Relax
(110)	3.87	5.47	90.0	21.2	29.0	0.600	27	44.9	37.9
(111)	3.87	3.87	60.0	13.0	31.6	0.600	18	21.1	21.1
(211)	3.87	9.47	90.0	36.6	27.9	0.560	42	71.2	49.5
(310)	5.47	8.65	90.0	47.3	27.7	0.563	54	107.8	77.1
(311)	3.87	6.70	73.2	24.8	31.3	0.579	33	89.1	59.2
(331)	3.87	8.65	77.1	32.6	28.9	0.565	39	38.3	32.0

Table S5. Geometry of K_2O slabs.

Termination	a (Å)	b (Å)	γ (°)	Area A (Å^2)	Height (Å)	Occupancy	#atoms/ cell	E_{surf} (meV/ Å^2) Fix	Relax
(110)	4.54	6.42	90.0	29.1	29.5	0.615	24	25.7	20.1
(111)	4.54	4.54	60.0	17.8	33.3	0.667	18	11.8	11.0
(211)	4.54	11.11	90.0	50.4	27.5	0.571	36	41.6	23.9
(310)	6.42	10.14	90.0	65.1	27.4	0.593	48	64.9	38.0
(311)	4.54	7.86	73.2	34.1	29.0	0.600	27	52.2	28.7
(331)	4.54	10.14	77.1	44.9	30.9	0.619	39	21.6	16.3

Table S6. Geometry of rutile TiO_2 slabs. The * behind the termination indicates stability against macroscopic facet formation.

Termination	*a* (Å)	*b* (Å)	*γ* (°)	Area *A* (Å^2)	Height (Å)	Occupancy	#atoms/ cell	E_{surf} (meV/ Å^2)	
								Fix	Relax
(001)	4.64	4.64	90.0	21.5	27.0	0.611	33	152.0	103.0
(100)*	3.00	4.64	90.0	13.9	37.1	0.562	27	91.9	58.1
(101)*	4.64	5.53	90.0	25.6	32.8	0.615	48	106.1	76.7
(102)	4.64	9.75	90.0	45.2	31.4	0.591	78	276.4	135.5
(110)*	3.00	6.56	90.0	19.7	32.8	0.600	36	85.2	45.3
(111)	5.53	6.56	126.4	29.2	33.2	0.633	57	172.5	105.9
(112)	6.56	7.21	90.0	47.3	31.4	0.609	84	255.7	171.1
(201)	4.64	7.59	90.0	35.2	27.5	0.567	51	135.8	79.3
(211)	5.53	7.21	71.7	37.8	32.4	0.579	66	129.7	77.8
(221)A	6.56	7.59	64.4	44.9	31.7	0.614	81	215.5	132.9
(221)B*	6.56	7.59	64.4	44.9	31.7	0.614	81	153.1	70.1
(301)	4.64	10.13	90.0	47.0	28.9	0.571	72	138.3	79.6
(310)	3.00	14.67	90.0	44.0	32.3	0.591	78	229.4	91.6
(320)A	3.00	16.73	90.0	50.2	30.9	0.604	87	210.7	90.6
(320)B	3.00	16.73	90.0	50.2	30.9	0.604	87	234.4	115.2
(321)*	7.21	7.59	93.7	54.6	31.9	0.593	96	122.5	68.4

Table S7. Geometry of SnO_2 slabs. The * behind the termination indicates stability against macroscopic facet formation.

Termination	a (Å)	b (Å)	γ (°)	Area A (Å^2)	Height (Å)	Occupancy	#atoms/ cell	E_{surf} (meV/ Å^2)	
								Fix	Relax
(001)	4.78	4.78	90.0	22.8	28.9	0.556	30	152.2	122.0
(100)*	3.21	4.78	90.0	15.4	38.2	0.563	27	90.8	72.8
(101)*	4.78	5.76	90.0	27.5	32.0	0.583	42	117.1	93.6
(102)	4.78	10.08	90.0	48.2	30.5	0.600	72	214.5	159.6
(110)*	3.21	6.76	90.0	21.7	33.8	0.600	36	88.4	65.4
(111)	5.76	6.76	125.9	31.5	32.6	0.607	51	175.6	134.7
(112)	6.76	7.48	90.0	50.6	31.9	0.591	78	192.8	165.7
(201)	4.78	8.01	90.0	38.3	28.8	0.567	51	131.0	95.7
(211)*	5.76	7.48	73.1	41.2	32.0	0.611	66	128.9	94.4
(221)A	6.76	8.01	65.1	49.1	31.4	0.595	75	154.5	140.8
(221)B*	6.76	8.01	65.1	49.1	32.9	0.614	81	143.8	89.7
(301)*	4.78	10.76	90.0	51.4	28.5	0.600	72	129.2	91.5
(310)	3.21	15.11	90.0	48.6	30.2	0.600	72	169.4	156.2
(320)A	3.21	17.23	90.0	55.4	31.8	0.604	87	171.8	115.0
(320)B	3.21	17.23	90.0	55.4	31.8	0.604	87	166.5	114.0
(321)*	7.48	8.01	92.1	59.9	30.6	0.600	90	122.9	89.1

Table S8. Geometry of GeO_2 slabs. The * behind the termination indicates stability against macroscopic facet formation.

Termination	a (Å)	b (Å)	γ (°)	Area A (Å^2)	Height (Å)	Occupancy	#atoms/ cell	E_{surf} (meV/ Å^2)	
								Fix	Relax
(001)	4.45	4.45	90.0	19.8	29.1	0.550	33	185.1	131.3
(100)*	2.91	4.45	90.0	13.0	35.6	0.563	27	90.5	57.4
(101)*	4.45	5.32	90.0	23.7	31.7	0.615	48	136.7	94.5
(102)	4.45	9.36	90.0	41.7	31.8	0.587	81	242.8	182.3
(110)*	2.91	6.29	90.0	18.3	31.5	0.600	36	95.4	56.2
(111)	5.32	6.29	126.3	27.0	32.1	0.633	57	200.9	136.8
(112)	6.29	6.93	90.0	43.6	31.7	0.583	84	221.8	181.6
(201)	4.45	7.33	90.0	32.6	28.3	0.563	54	147.3	89.4
(211)*	5.32	6.93	72.1	35.1	31.2	0.579	66	147.0	86.7
(221)A	6.29	7.33	64.6	41.6	31.8	0.587	81	173.9	153.4
(221)B*	6.29	7.33	64.6	41.6	31.8	0.630	87	161.7	84.5
(301)*	4.45	9.80	90.0	43.6	27.8	0.571	72	143.9	79.8
(310)	2.91	14.07	90.0	41.0	31.0	0.591	78	186.2	158.8
(320)A	2.91	16.05	90.0	46.7	32.1	0.596	93	189.6	95.0
(320)B	2.91	16.05	90.0	46.7	32.1	0.596	93	169.5	143.5
(321)*	6.93	7.33	93.2	50.7	30.7	0.593	96	137.4	79.8

Table S9. Geometry of anatase TiO_2 slabs. The * behind the termination indicates stability against macroscopic facet formation.

Termination	*a* (Å)	*b* (Å)	*γ* (°)	Area *A* ($Å^2$)	Height (Å)	Occupancy	#atoms/ cell	E_{surf} (meV/ $Å^2$)	
								Fix	Relax
(001)*	3.86	3.86	90.0	14.9	33.4	0.571	24	75.5	73.4
(100)	3.86	9.56	90.0	36.9	28.9	0.600	54	91.4	47.9
(101)A*	3.86	5.50	69.5	19.9	35.8	0.600	36	70.1	38.7
(101)B	3.86	5.50	69.5	19.9	35.8	0.600	36	131.5	90.5
(102)	3.86	12.28	90.0	47.4	28.5	0.579	66	209.0	124.1
(103)A	3.86	7.75	75.6	28.9	31.9	0.615	48	141.6	73.7
(103)B	3.86	7.75	75.6	28.9	31.9	0.615	48	198.2	68.2
(110)	5.46	5.50	119.7	26.1	27.3	0.600	36	128.7	80.3
(111)	5.46	10.31	74.7	54.2	27.5	0.571	72	239.1	118.5
(112)	5.46	5.50	90.0	30.0	33.2	0.571	48	89.6	58.0
(114)	5.46	7.75	69.4	39.6	32.4	0.583	63	232.4	73.7
(211)A	5.50	8.63	118.1	41.9	32.3	0.579	66	241.2	152.9
(211)B	5.50	8.63	118.1	41.9	32.3	0.579	66	111.7	68.6
(213)A	5.50	8.63	81.0	46.9	30.3	0.600	72	270.8	74.2
(213)B	5.50	8.63	81.0	46.9	30.3	0.600	72	162.2	96.2
(301)A	3.86	14.59	82.4	55.8	31.9	0.600	90	86.5	45.0
(301)B	3.86	14.59	82.4	55.8	31.9	0.600	90	107.8	63.5

Table S10. Geometry of cleaved Ga_2O_3 slabs. The surface energy is when atom positions were fixed to that of cleaved bulk.

Termination	*a* (Å)	*b* (Å)	*γ* (°)	Area *A* (Å^2)	Height (Å)	Occu-pancy	#atoms/ cell	E_{surf} (meV/ Å^2)
(001)A	3.07	6.36	104.0	19.0	45.6	0.625	50	135.2
(001)B	3.07	6.36	104.0	19.0	45.6	0.625	50	155.0
(010)	5.86	12.35	103.7	70.3	27.6	0.611	110	143.9
(100)A	3.07	5.86	90.0	18.0	48.0	0.625	50	41.3
(100)B	3.07	5.86	90.0	18.0	48.0	0.625	50	84.8
(10 $\bar{1}$)A	3.07	12.35	90.0	37.9	34.2	0.583	70	197.4
(101)A	3.07	14.88	90.0	45.7	33.1	0.571	80	283.6
(10 $\bar{1}$)B	3.07	12.35	90.0	37.9	34.2	0.583	70	175.3
(101)B	3.07	14.88	90.0	45.7	33.1	0.571	80	186.6
(110)A	5.86	6.36	103.3	36.3	35.7	0.583	70	218.8
(110)B	5.86	6.36	103.3	36.3	35.7	0.583	70	157.0
(11 $\bar{1}$)A	6.36	7.59	124.2	40.0	32.4	0.583	70	213.1
(111)A	6.36	6.62	84.7	41.9	30.9	0.583	70	115.3
(11 $\bar{1}$)B	6.36	7.59	124.2	40.0	32.4	0.583	70	120.8
(111)B	6.36	6.62	84.7	41.9	30.9	0.583	70	258.5
(11 $\bar{2}$)A	6.36	8.75	113.8	50.9	31.8	0.600	90	152.8
(112)A	6.36	10.53	53.7	54.0	34.0	0.588	100	174.3
(11 $\bar{2}$)B	6.36	8.75	113.8	50.9	31.8	0.600	90	306.8
(112)B	6.36	10.53	53.7	54.0	34.0	0.588	100	136.3
(11 $\bar{3}$)A	6.36	10.69	105.1	65.7	31.3	0.579	110	169.0
(113)A	6.36	12.19	63.2	69.3	31.2	0.600	120	196.8
(11 $\bar{3}$)B	6.36	10.69	105.1	65.7	31.3	0.579	110	152.7
(113)B	6.36	12.19	63.2	69.3	31.2	0.600	120	172.5
(20 $\bar{1}$)A	3.07	7.59	78.3	22.8	37.8	0.625	50	135.8
(201)A	3.07	9.60	80.8	29.1	37.1	0.600	60	232.4
(20 $\bar{1}$)B	3.07	7.59	78.3	22.8	37.8	0.625	50	230.0
(201)B	3.07	9.60	80.8	29.1	37.1	0.600	60	147.9
(20 $\bar{3}$)A	3.07	18.12	85.1	55.5	33.1	0.588	100	185.0
(203)A	3.07	20.77	85.8	63.6	32.3	0.579	110	268.0
(20 $\bar{3}$)B	3.07	18.12	85.1	55.5	33.1	0.588	100	175.6
(203)B	3.07	20.77	85.8	63.6	32.3	0.579	110	208.3
(30 $\bar{1}$)A	3.07	18.94	90.0	58.2	31.6	0.588	100	178.5
(30 $\bar{1}$)B	3.07	18.94	90.0	58.2	31.6	0.588	100	184.6

(310)A	5.86	7.70	101.0	44.3	31.7	0.615	80	284.7
(310)B	5.86	7.70	101.0	44.3	31.7	0.615	80	178.1
$(31\bar{1})$A	6.62	7.70	116.5	45.6	33.1	0.571	80	240.4
(311)A	6.62	7.70	83.8	50.7	32.0	0.600	90	202.4
$(31\bar{1})$B	6.62	7.70	116.5	45.6	33.1	0.571	80	146.8
(311)B	6.62	7.70	83.8	50.7	32.0	0.600	90	144.8
$(31\bar{2})$A	7.59	7.70	67.4	54.0	34.0	0.588	100	182.3
(312)A	7.70	9.60	57.6	62.4	31.2	0.611	110	146.2
$(31\bar{2})$B	7.59	7.70	67.4	54.0	34.0	0.588	100	184.2
(312)B	7.70	9.60	57.6	62.4	31.2	0.611	110	139.9
$(31\bar{3})$A	7.70	8.75	97.1	66.9	32.3	0.600	120	153.2
$(31\bar{3})$B	7.70	8.75	97.1	66.9	32.3	0.600	120	196.0
$(40\bar{1})$A	3.07	11.98	82.6	36.5	35.5	0.583	70	294.0
(401)A	3.07	14.57	84.0	44.5	31.6	0.615	80	120.3
$(40\bar{1})$B	3.07	11.98	82.6	36.5	35.5	0.583	70	115.2
(401)B	3.07	14.57	84.0	44.5	31.6	0.615	80	236.7
$(40\bar{3})$A	3.07	19.49	85.5	59.7	30.8	0.588	100	190.3
$(40\bar{3})$B	3.07	19.49	85.5	59.7	30.8	0.588	100	164.2
(510)A	5.86	9.85	98.6	57.1	32.1	0.588	100	264.1
(510)B	5.86	9.85	98.6	57.1	32.1	0.588	100	180.1
$(51\bar{1})$A	6.62	9.85	119.6	56.7	32.4	0.588	100	158.3
(511)A	6.62	9.85	76.7	63.5	32.3	0.579	110	189.4
$(51\bar{1})$B	6.62	9.85	119.6	56.7	32.4	0.588	100	191.1
(511)B	6.62	9.85	76.7	63.5	32.3	0.579	110	136.5
$(51\bar{2})$A	7.59	8.49	104.7	62.4	31.2	0.611	110	129.7
$(51\bar{2})$B	7.59	8.49	104.7	62.4	31.2	0.611	110	196.6
$(60\bar{1})$A	3.07	17.27	84.9	52.8	30.7	0.600	90	265.8
(601)A	3.07	20.04	85.6	61.3	31.7	0.611	110	208.6
$(60\bar{1})$B	3.07	17.27	84.9	52.8	30.7	0.600	90	102.1
(601)B	3.07	20.04	85.6	61.3	31.7	0.611	110	184.9
$(71\bar{1})$A	6.62	12.40	120.5	70.7	30.6	0.600	120	166.7
$(71\bar{1})$B	6.62	12.40	120.5	70.7	30.6	0.600	120	197.3
$(80\bar{1})$A	3.07	22.84	86.1	70.0	30.9	0.600	120	164.3
$(80\bar{1})$B	3.07	22.84	86.1	70.0	30.9	0.600	120	198.8

Table S11. Geometry of cleaved Al_2O_3 slabs. The surface energy is when atom positions were fixed to that of cleaved bulk.

Termination	a (Å)	b (Å)	γ (°)	Area A (Å^2)	Height (Å)	Occu-pancy	#atoms/cell	E_{surf} (meV/Å^2)
(001)A	2.92	6.08	103.9	17.2	43.7	0.625	50	182.6
(001)B	2.92	6.08	103.9	17.2	43.7	0.625	50	198.8
(010)	5.64	11.81	104.1	64.6	27.7	0.579	110	183.3
(100)A	2.92	5.64	90.0	16.5	45.8	0.625	50	45.8
(100)B	2.92	5.64	90.0	16.5	45.8	0.625	50	104.9
$(10\bar{1})$A	2.92	11.79	90.0	34.4	32.9	0.583	70	266.7
(101)A	2.92	14.27	90.0	41.7	31.7	0.571	80	393.5
$(10\bar{1})$B	2.92	11.79	90.0	34.4	32.9	0.583	70	236.8
(101)B	2.92	14.27	90.0	41.7	31.7	0.571	80	249.7
(110)A	5.64	6.08	103.6	33.3	34.0	0.583	70	289.2
(110)B	5.64	6.08	103.6	33.3	34.0	0.583	70	203.6
$(11\bar{1})$A	6.08	7.25	124.0	36.6	30.9	0.583	70	284.0
(111)A	6.08	6.35	84.3	38.4	31.9	0.615	80	140.0
$(11\bar{1})$B	6.08	7.25	124.0	36.6	30.9	0.583	70	151.6
(111)B	6.08	6.35	84.3	38.4	31.9	0.615	80	338.3
$(11\bar{2})$A	6.08	8.35	113.7	46.5	30.4	0.600	90	206.2
(112)A	6.08	10.10	53.5	49.4	32.5	0.588	100	232.2
$(11\bar{2})$B	6.08	8.35	113.7	46.5	30.4	0.600	90	413.6
(112)B	6.08	10.10	53.5	49.4	32.5	0.588	100	169.5
$(11\bar{3})$A	6.08	10.19	105.1	59.8	31.5	0.600	120	224.7
(113)A	6.08	11.67	62.9	63.2	32.8	0.591	130	319.4
$(11\bar{3})$B	6.08	10.19	105.1	59.8	31.5	0.600	120	201.0
(113)B	6.08	11.67	62.9	63.2	32.8	0.591	130	224.6
$(20\bar{1})$A	2.92	7.25	78.4	20.7	36.4	0.625	50	182.9
(201)A	2.92	9.22	80.9	26.6	35.5	0.600	60	323.5
$(20\bar{1})$B	2.92	7.25	78.4	20.7	36.4	0.625	50	351.1
(201)B	2.92	9.22	80.9	26.6	35.5	0.600	60	191.3
$(20\bar{3})$A	2.92	17.30	85.2	50.3	31.8	0.588	100	249.6
(203)A	2.92	19.91	85.8	58.0	32.5	0.600	120	362.2
$(20\bar{3})$B	2.92	17.30	85.2	50.3	31.8	0.588	100	249.4
(203)B	2.92	19.91	85.8	58.0	32.5	0.600	120	304.1
$(30\bar{1})$A	2.92	18.12	90.0	52.9	32.1	0.611	110	239.7
$(30\bar{1})$B	2.92	18.12	90.0	52.9	32.1	0.611	110	261.7

(310)A	5.64	7.35	101.3	40.6	32.5	0.571	80	308.8
(310)B	5.64	7.35	101.3	40.6	32.5	0.571	80	241.2
$(31\bar{1})$A	6.35	7.35	116.6	41.7	31.6	0.571	80	323.8
(311)A	6.35	7.35	84.2	46.4	30.5	0.600	90	279.0
$(31\bar{1})$B	6.35	7.35	116.6	41.7	31.6	0.571	80	190.6
(311)B	6.35	7.35	84.2	46.4	30.5	0.600	90	189.6
$(31\bar{2})$A	7.25	7.35	67.5	49.3	32.5	0.588	100	244.6
(312)A	7.35	9.22	57.3	57.1	31.4	0.579	110	189.6
$(31\bar{2})$B	7.25	7.35	67.5	49.3	32.5	0.588	100	249.6
(312)B	7.35	9.22	57.3	57.1	31.4	0.579	110	247.2
$(31\bar{3})$A	7.35	8.35	97.1	60.9	31.0	0.600	120	201.2
$(31\bar{3})$B	7.35	8.35	97.1	60.9	31.0	0.600	120	258.7
$(40\bar{1})$A	2.92	11.48	82.7	33.2	34.0	0.583	70	391.6
(401)A	2.92	14.01	84.0	40.7	32.4	0.571	80	154.6
$(40\bar{1})$B	2.92	11.48	82.7	33.2	34.0	0.583	70	150.1
(401)B	2.92	14.01	84.0	40.7	32.4	0.571	80	317.0
$(40\bar{3})$A	2.92	18.60	85.5	54.2	31.3	0.611	110	254.1
$(40\bar{3})$B	2.92	18.60	85.5	54.2	31.3	0.611	110	325.6
(510)A	5.64	9.39	98.8	52.3	30.6	0.588	100	359.7
(510)B	5.64	9.39	98.8	52.3	30.6	0.588	100	245.0
$(51\bar{1})$A	6.35	9.39	119.6	51.8	30.9	0.588	100	210.2
(511)A	6.35	9.39	77.2	58.1	32.4	0.600	120	250.1
$(51\bar{1})$B	6.35	9.39	119.6	51.8	30.9	0.588	100	253.4
(511)B	6.35	9.39	77.2	58.1	32.4	0.600	120	177.8
$(51\bar{2})$A	7.25	8.12	104.9	56.9	31.5	0.579	110	164.6
$(51\bar{2})$B	7.25	8.12	104.9	56.9	31.5	0.579	110	268.0
$(60\bar{1})$A	2.92	16.56	84.9	48.2	33.3	0.588	100	360.6
(601)A	2.92	19.27	85.7	56.1	31.9	0.579	110	277.9
$(60\bar{1})$B	2.92	16.56	84.9	48.2	33.3	0.588	100	131.7
(601)B	2.92	19.27	85.7	56.1	31.9	0.579	110	247.2
$(71\bar{1})$A	6.35	11.80	120.4	64.6	32.1	0.591	130	218.9
$(71\bar{1})$B	6.35	11.80	120.4	64.6	32.1	0.591	130	261.7
$(80\bar{1})$A	2.92	21.92	86.2	63.9	32.5	0.591	130	217.1
$(80\bar{1})$B	2.92	21.92	86.2	63.9	32.5	0.591	130	253.2

Table S12. Geometry of cleaved ZrO_2 slabs. The surface energy is when atom positions were fixed to that of cleaved bulk.

Termination	a (Å)	b (Å)	γ (°)	Area A (Å^2)	Height (Å)	Occu-pancy	#atoms/ cell	E_{surf} (meV/ Å^2)
(001)A	5.20	5.25	90.0	27.3	36.9	0.643	54	221.4
(001)B	5.20	5.25	90.0	27.3	36.9	0.643	54	143.2
(01$\bar{2}$)A	5.20	11.78	85.7	61.1	33.0	0.607	102	182.9
(01$\bar{2}$)B	5.20	11.78	85.7	61.1	33.0	0.607	102	167.6
(01$\bar{2}$)C	5.20	11.78	85.7	61.1	33.0	0.571	96	219.3
(01$\bar{2}$)D	5.20	11.78	85.7	61.1	33.0	0.571	96	158.8
(01$\bar{1}$)A	5.20	7.49	83.2	38.7	37.2	0.600	72	152.9
(01$\bar{1}$)B	5.20	7.49	83.2	38.7	37.2	0.600	72	130.4
(010)A	5.20	5.35	80.5	27.5	36.7	0.643	54	176.2
(010)B	5.20	5.35	80.5	27.5	36.7	0.643	54	238.1
(011)A	5.20	7.49	83.2	38.7	37.2	0.600	72	152.9
(011)B	5.20	7.49	83.2	38.7	37.2	0.600	72	130.4
(012)A	5.20	11.78	85.7	61.1	33.0	0.607	102	167.6
(012)B	5.20	11.78	85.7	61.1	33.0	0.607	102	182.9
(012)C	5.20	11.78	85.7	61.1	33.0	0.571	96	158.8
(012)D	5.20	11.78	85.7	61.1	33.0	0.571	96	219.3
(02$\bar{1}$)A	5.20	11.92	81.5	61.3	32.9	0.571	96	222.1
(02$\bar{1}$)B	5.20	11.92	81.5	61.3	32.9	0.571	96	227.0
(021)A	5.20	11.92	81.5	61.3	32.9	0.571	96	227.0
(021)B	5.20	11.92	81.5	61.3	32.9	0.571	96	222.1
(10$\bar{2}$)A	5.25	10.89	90.0	57.1	32.8	0.615	96	192.3
(10$\bar{2}$)B	5.25	10.89	90.0	57.1	32.8	0.615	96	187.4
(10$\bar{1}$)A	5.25	6.82	90.0	35.8	32.2	0.625	60	122.3
(10$\bar{1}$)B	5.25	6.82	90.0	35.8	32.2	0.625	60	248.6
(100)A	5.25	5.35	90.0	28.1	41.1	0.625	60	137.2
(100)B	5.25	5.35	90.0	28.1	41.1	0.625	60	237.0
(101)A	5.25	8.06	90.0	42.3	30.7	0.556	60	152.8
(101)B	5.25	8.06	90.0	42.3	34.1	0.550	66	181.7
(102)A	5.25	12.46	90.0	65.4	33.1	0.600	108	125.1
(102)B	5.25	12.46	90.0	65.4	33.1	0.600	108	211.0
(11$\bar{2}$)A	7.39	10.89	128.0	63.4	31.8	0.607	102	205.4
(11$\bar{2}$)B	7.39	10.89	128.0	63.4	31.8	0.607	102	233.0
(11$\bar{2}$)C	7.39	10.89	128.0	63.4	31.8	0.571	96	244.6

$(11\bar{2})$D	7.39	10.89	128.0	63.4	31.8	0.571	96	197.5
$(11\bar{1})$A	6.82	7.39	63.5	45.1	31.9	0.600	72	93.6
$(11\bar{1})$B	6.82	7.39	63.5	45.1	31.9	0.600	72	246.9
(110)A	5.35	7.39	96.7	39.3	36.7	0.600	72	157.7
(110)B	5.35	7.39	96.7	39.3	36.7	0.600	72	127.1
(111)A	7.39	8.06	57.9	50.4	34.3	0.583	84	262.4
(111)B	7.39	8.06	57.9	50.4	34.3	0.583	84	100.2
(112)A	7.39	9.61	86.6	70.9	30.5	0.600	108	188.5
(112)B	7.39	9.61	86.6	70.9	30.5	0.600	108	317.4
$(12\bar{2})$A	10.89	11.65	38.6	79.2	30.9	0.588	120	263.7
$(12\bar{2})$B	10.89	11.65	38.6	79.2	30.9	0.588	120	187.1
$(12\bar{1})$A	6.82	11.65	55.6	65.6	33.0	0.600	108	173.9
$(12\bar{1})$B	6.82	11.65	55.6	65.6	33.0	0.600	108	309.1
(120)A	5.35	11.65	98.5	61.7	32.7	0.607	102	247.7
(120)B	5.35	11.65	98.5	61.7	32.7	0.607	102	222.6
(120)C	5.35	11.65	98.5	61.7	32.7	0.571	96	234.0
(120)D	5.35	11.65	98.5	61.7	32.7	0.571	96	166.3
(121)A	8.06	11.65	132.4	69.3	33.3	0.594	114	187.5
(121)B	8.06	11.65	132.4	69.3	33.3	0.594	114	189.5
(121)C	8.06	11.65	132.4	69.3	31.2	0.600	108	255.7
(121)D	8.06	11.65	132.4	69.3	31.2	0.600	108	250.6
$(20\bar{1})$A	5.25	11.10	90.0	58.2	32.2	0.577	90	162.3
$(20\bar{1})$B	5.25	11.10	90.0	58.2	32.2	0.577	90	176.1
(201)A	5.25	12.65	90.0	66.4	32.6	0.567	102	259.3
(201)B	5.25	12.65	90.0	66.4	32.6	0.567	102	248.7
$(21\bar{2})$A	6.82	11.71	73.7	76.6	32.0	0.588	120	181.2
$(21\bar{2})$B	6.82	11.71	73.7	76.6	32.0	0.588	120	246.8
$(21\bar{1})$A	7.49	8.60	93.1	64.4	33.6	0.600	108	136.6
$(21\bar{1})$B	7.49	8.60	93.1	64.4	33.6	0.600	108	213.0
(210)A	5.35	11.71	94.2	62.5	32.3	0.571	96	221.9
(210)B	5.35	11.71	94.2	62.5	32.3	0.571	96	290.5
(211)A	7.49	9.61	94.6	71.8	30.1	0.600	108	214.7
(211)B	7.49	9.61	94.6	71.8	30.1	0.600	108	225.9
$(22\bar{1})$A	7.39	11.10	102.6	80.1	32.4	0.611	132	191.1
$(22\bar{1})$B	7.39	11.10	102.6	80.1	32.4	0.611	132	221.5

Table S13. Geometry of cleaved ZrO_2 slabs used to check facet decomposition. Atom positions were relaxed. The * behind the termination indicates stability against macroscopic facet formation, and ** indicates surfaces that could not be converged.

Termination	a (Å)	b (Å)	γ (°)	Area A (Å^2)	Height (Å)	Occu-pancy	#atoms/ cell	E_{surf} (meV/ Å^2)
(001)A	5.20	5.25	90.0	27.3	36.9	0.643	54	146.1
(001)B*	5.20	5.25	90.0	27.3	36.9	0.643	54	105.7
$(01\bar{2})$A	5.20	11.78	85.7	61.1	33.0	0.607	102	111.6
$(01\bar{2})$B*	5.20	11.78	85.7	61.1	33.0	0.607	102	104.8
$(01\bar{2})$C	5.20	11.78	85.7	61.1	33.0	0.571	96	133.9
$(01\bar{2})$D	5.20	11.78	85.7	61.1	33.0	0.571	96	108.8
$(01\bar{1})$A	5.20	7.49	83.2	38.7	37.2	0.600	72	117.9
$(01\bar{1})$B	5.20	7.49	83.2	38.7	37.2	0.6	72	96.9
(010)A	5.20	5.35	80.5	27.5	36.7	0.643	54	125.9
(010)B	5.20	5.35	80.5	27.5	36.7	0.643	54	163.3
(011)A	5.20	7.49	83.2	38.7	37.2	0.600	72	117.9
(011)B	5.20	7.49	83.2	38.7	37.2	0.600	72	96.9
(012)A*	5.20	11.78	85.7	61.1	33.0	0.607	102	104.8
(012)B	5.20	11.78	85.7	61.1	28.3	0.542	78	110.3
(012)C	5.20	11.78	85.7	61.1	33.0	0.571	96	108.8
(012)D	5.20	11.78	85.7	61.1	33.0	0.571	96	133.9
$(02\bar{1})$A	5.20	11.92	81.5	61.3	30.5	0.538	84	147.9
$(02\bar{1})$B	5.20	11.92	81.5	61.3	30.5	0.538	84	122.8
(021)A	**	**	**	**	**	**	**	**
(021)B	5.20	11.92	81.5	61.3	32.9	0.571	96	147.7
$(10\bar{2})$A*	5.25	10.89	90.0	57.1	32.8	0.615	96	97.2
$(10\bar{2})$B	5.25	10.89	90.0	57.1	32.8	0.615	96	110.3
$(10\bar{1})$A	5.25	6.82	90.0	35.8	32.2	0.625	60	92.6
$(10\bar{1})$B	5.25	6.82	90.0	35.8	32.2	0.625	60	133.9
(100)A*	5.25	5.35	90.0	28.1	41.1	0.625	60	108.9
(100)B	5.25	5.35	90.0	28.1	41.1	0.625	60	145.9
(101)A	5.25	8.06	90.0	42.3	30.7	0.556	60	107.6
(101)B	5.25	8.06	90.0	42.3	34.1	0.550	66	124.0
(102)A*	5.25	12.46	90.0	65.4	33.1	0.600	108	92.7
(102)B	**	**	**	**	**	**	**	**
$(11\bar{2})$A	7.39	10.89	128.0	63.4	31.8	0.607	102	105.2
$(11\bar{2})$B	**	**	**	**	**	**	**	**

(11 $\bar{2}$)C*	7.39	10.89	128.0	63.4	31.8	0.571	96	94.0
(11 $\bar{2}$)D	**	**	**	**	**	**	**	**
(11 $\bar{1}$)A*	6.82	7.39	63.5	45.1	31.9	0.600	72	71.2
(11 $\bar{1}$)B	6.82	7.39	63.5	45.1	31.9	0.600	72	147.9
(110)A	5.35	7.39	96.7	39.3	36.7	0.600	72	114.9
(110)B*	5.35	7.39	96.7	39.3	36.7	0.600	72	93.1
(111)A	7.39	8.06	57.9	50.4	34.3	0.583	84	136.1
(111)B*	7.39	8.06	57.9	50.4	34.3	0.583	84	83.7
(112)A	7.39	9.61	86.6	70.9	30.5	0.600	108	116.2
(112)B	7.39	9.61	86.6	70.9	30.5	0.600	108	126.8
(12 $\bar{2}$)A	**	**	**	**	**	**	**	**
(12 $\bar{2}$)B	10.89	11.65	38.6	79.2	30.9	0.588	120	111.1
(12 $\bar{1}$)A	6.82	11.65	55.6	65.6	33.0	0.600	108	112.6
(12 $\bar{1}$)B	**	**	**	**	**	**	**	**
(120)A	5.35	11.65	98.5	61.7	32.7	0.607	102	113.8
(120)B	5.35	11.65	98.5	61.7	28.0	0.542	78	135.9
(120)C	5.35	11.65	98.5	61.7	32.7	0.571	96	153.0
(120)D*	5.35	11.65	98.5	61.7	32.7	0.571	96	111.6
(121)A	8.06	11.65	132.4	69.3	33.3	0.594	114	114.1
(121)B	8.06	11.65	132.4	69.3	33.3	0.594	114	119.0
(121)C	8.06	11.65	132.4	69.3	29.1	0.571	96	125.6
(121)D	8.06	11.65	132.4	69.3	31.2	0.600	108	146.7
(20 $\bar{1}$)A	5.25	11.10	90.0	58.2	32.2	0.577	90	104.5
(20 $\bar{1}$)B	5.25	11.10	90.0	58.2	32.2	0.577	90	109.4
(201)A	5.25	12.65	90.0	66.4	32.6	0.567	102	121.8
(201)B	**	**	**	**	**	**	**	**
(21 $\bar{2}$)A	6.82	11.71	73.7	76.6	28.2	0.533	96	112.2
(21 $\bar{2}$)B	6.82	11.71	73.7	76.6	32.0	0.588	120	112.1
(21 $\bar{1}$)A*	7.49	8.60	93.1	64.4	33.6	0.600	108	91.2
(21 $\bar{1}$)B	7.49	8.60	93.1	64.4	29.1	0.538	84	110.6
(210)A	**	**	**	**	**	**	**	**
(210)B	5.35	11.71	94.2	62.5	32.3	0.571	96	113.0
(211)A	7.49	9.61	94.6	71.8	30.1	0.600	108	127.2
(211)B	7.49	9.61	94.6	71.8	28.1	0.571	96	129.4
(22 $\bar{1}$)A	**	**	**	**	**	**	**	**
(22 $\bar{1}$)B	7.39	11.10	102.6	80.1	34.2	0.632	144	130.6

Table S14 the five terminations, in ascending order, with the smallest σ, five lowest fixed E_{surf}, and five lowest relaxed E_{surf} for MgO

σ	E_{surf}	
	Fix	Relax
(100)	(100)	(100)
(310)	(310)	(310)
(210)	(210)	(210)
(110)	(110)	(110)
(211)	(211)	(221)

Table S15 the five terminations, in ascending order, with the smallest σ, five lowest fixed E_{surf}, and five lowest relaxed E_{surf} for CaO

σ	E_{surf}	
	Fix	Relax
(100)	(100)	(100)
(310)	(310)	(310)
(210)	(210)	(210)
(110)	(110)	(110)
(211)	(211)	(211)

Table S16 the five terminations, in ascending order, with the smallest σ, five lowest fixed E_{surf}, and five lowest relaxed E_{surf} for Li_2O

σ	E_{surf}	
	Fix	Relax
(111)	(111)	(111)
(331)	(331)	(331)
(110)	(110)	(110)
(211)	(211)	(211)
(311)	(311)	(311)

Table S17 the five terminations, in ascending order, with the smallest σ, five lowest fixed E_{surf}, and five lowest relaxed E_{surf} for Na_2O

σ	E_{surf}	
	Fix	Relax
(111)	(111)	(111)
(331)	(331)	(331)
(110)	(110)	(110)
(211)	(211)	(211)
(311)	(311)	(311)

Table S18 the five terminations, in ascending order, with the smallest σ, five lowest fixed E_{surf}, and five lowest relaxed E_{surf} for K_2O

σ	E_{surf}	
	Fix	Relax
(111)	(111)	(111)
(331)	(331)	(331)
(110)	(110)	(110)
(211)	(211)	(211)
(311)	(311)	(311)

Table S19 the five terminations, in ascending order, with the smallest σ, five lowest fixed E_{surf}, and five lowest relaxed E_{surf} for rutile TiO_2

σ	E_{surf}	
	Fix	Relax
(110)	(110)	(110)
(100)	(100)	(100)
(321)	(101)	(321)
(101)	(321)	(221)B
(211)	(211)	(101)

Table S20 the five terminations, in ascending order, with the smallest σ, five lowest fixed E_{surf}, and five lowest relaxed E_{surf} for SnO_2

σ	E_{surf}	
	Fix	Relax
(110)	(110)	(110)
(100)	(100)	(100)
(321)	(101)	(321)
(320)A	(321)	(221)B
(320)B	(211)	(301)

Table S21 the five terminations, in ascending order, with the smallest σ, five lowest fixed E_{surf}, and five lowest relaxed E_{surf} for GeO_2

σ	E_{surf}	
	Fix	Relax
(110)	(100)	(110)
(100)	(110)	(100)
(321)	(101)	(301)
(101)	(321)	(321)
(211)	(301)	(221)B

Table S22 the five terminations, in ascending order, with the smallest σ, five lowest fixed E_{surf}, and five lowest relaxed E_{surf} for anatase TiO_2

σ	E_{surf}	
	Fix	Relax
(101)A	(101)A	(101)A
(301)A	(001)	(301)A
(100)	(301)A	(100)
(112)	(112)	(112)
(001)	(100)	(103)B

Table S23 the five terminations, in ascending order, with the smallest σ, five lowest fixed E_{surf}, and five lowest relaxed E_{surf} for Ga_2O_3

σ	E_{surf}	
	Fix	Relax
$(20\bar{1})$A	(100)A	(100)A
(001)B	(100)B	$(20\bar{1})$A
$(40\bar{1})$B	$(60\bar{1})$B	$(11\bar{2})$A
(100)A	$(40\bar{1})$B	$(11\bar{3})$A
(100)B	(111)A	(100)B

Table S24 the five terminations, in ascending order, with the smallest σ, five lowest fixed E_{surf}, and five lowest relaxed E_{surf} for Al_2O_3

σ	E_{surf}	
	Fix	Relax
$(20\bar{1})$A	(100)A	(100)A
(001)B	(100)B	$(20\bar{1})$B
$(40\bar{1})$B	$(60\bar{1})$B	$(11\bar{2})$A
(100)A	(111)A	$(11\bar{3})$A
(100)B	$(40\bar{1})$B	$(20\bar{1})$A

Table S25 the five terminations, in ascending order, with the smallest σ, five lowest fixed E_{surf}, and five lowest relaxed E_{surf} for ZrO_2

σ	E_{surf}	
	Fix	Relax
$(11\bar{1})$A	$(11\bar{1})$A	$(11\bar{1})$A
(100)A	(111)B	(111)B
(110)B	$(10\bar{1})$A	$(21\bar{1})$A
$(21\bar{1})$A	(102)A	$(10\bar{1})$A
$(01\bar{1})$B	(110)B	(102)A

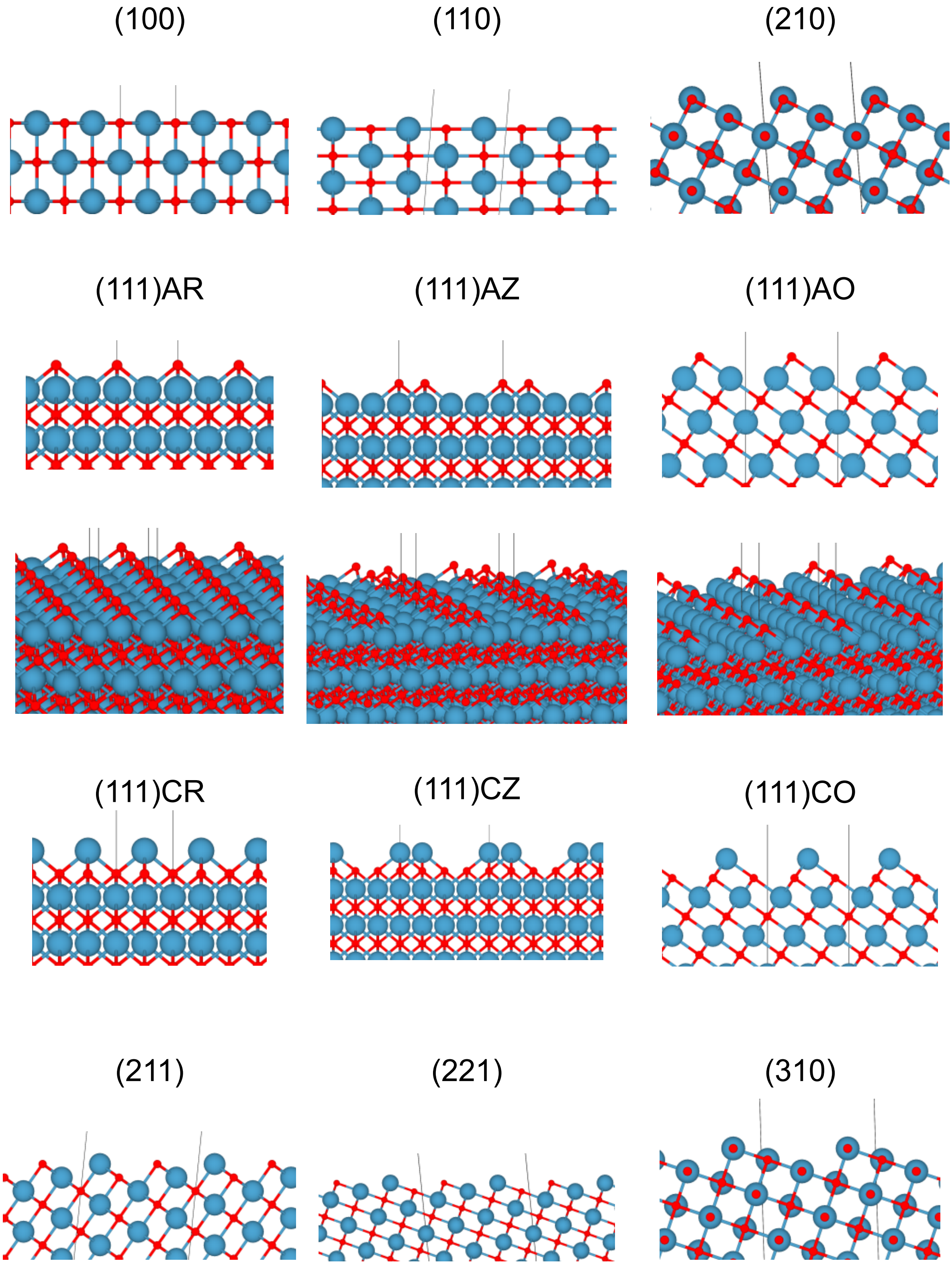

Figure S1. Terminations of unrelaxed rocksalt structure MgO and CaO slabs. Blue and red balls correspond to metal and O atoms, respectively.

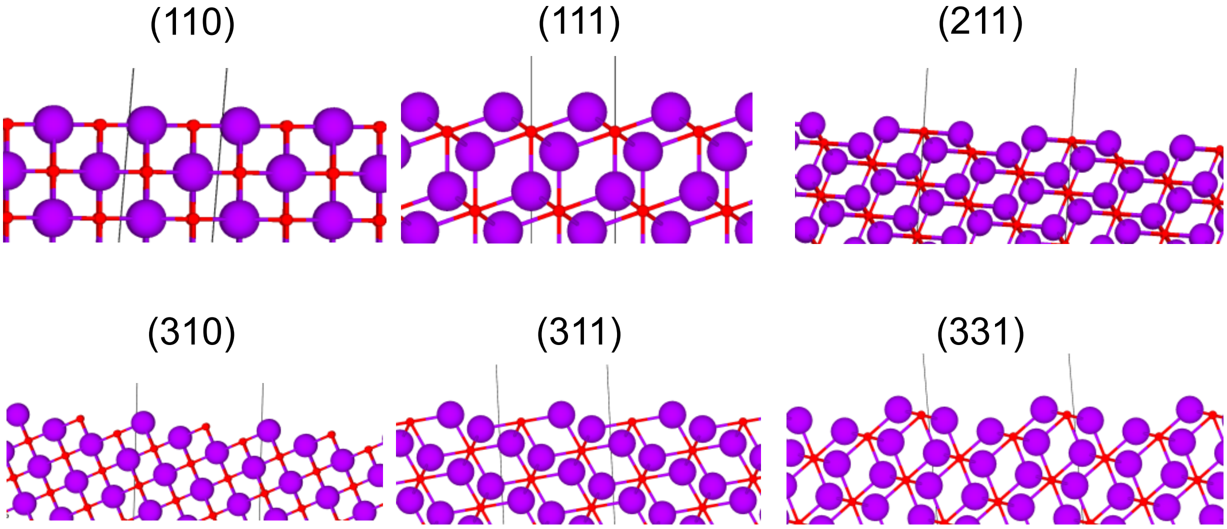

Figure S2. Terminations of cleaved antifluorite structure Li_2O, Na_2O, and K_2O slabs. Purple and red balls correspond to metal and O atoms, respectively.

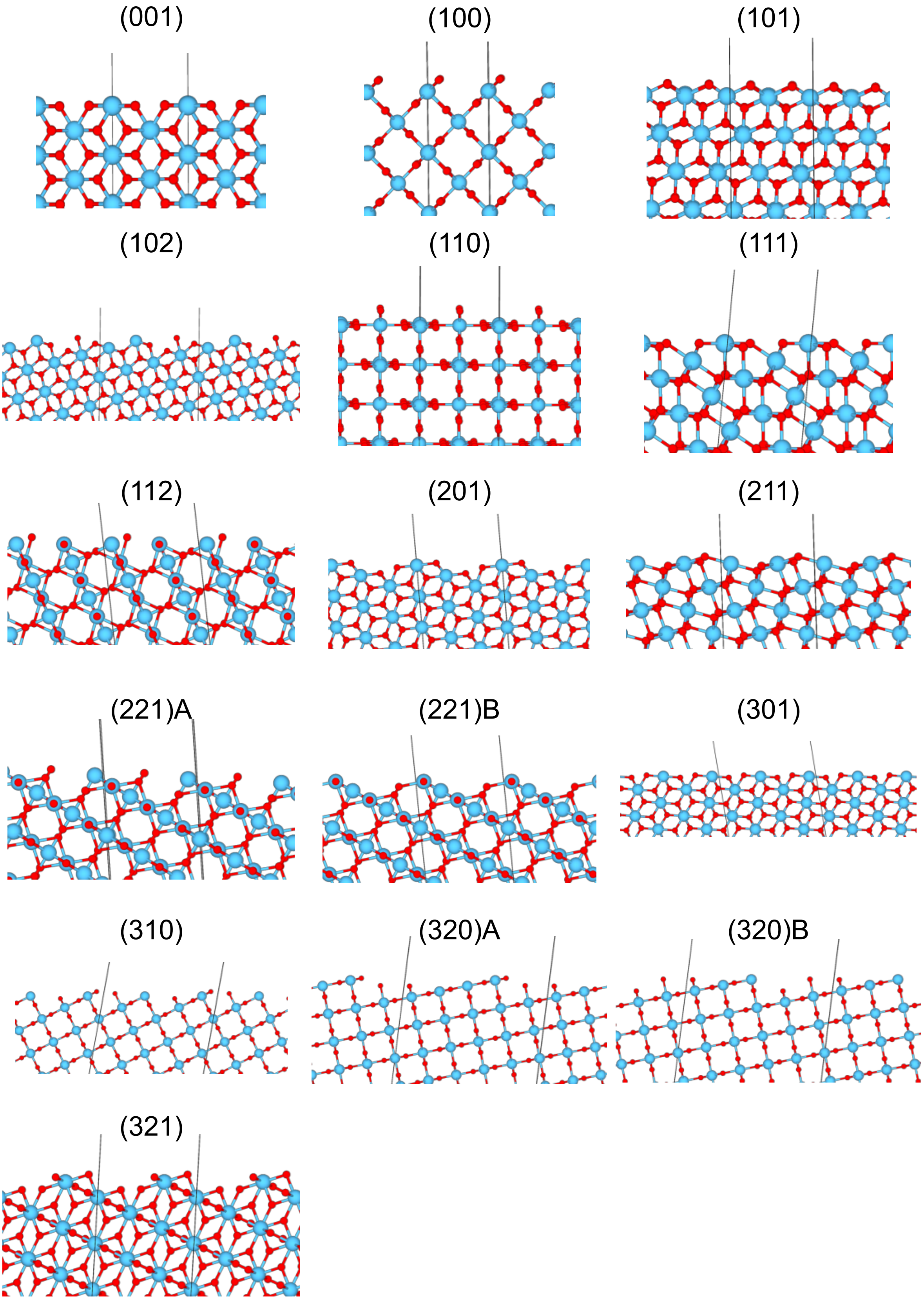

Figure S3. Terminations of cleaved rutile structure TiO_2, SnO_2, and GeO_2 slabs. Blue and red balls correspond to metal and O atoms, respectively.

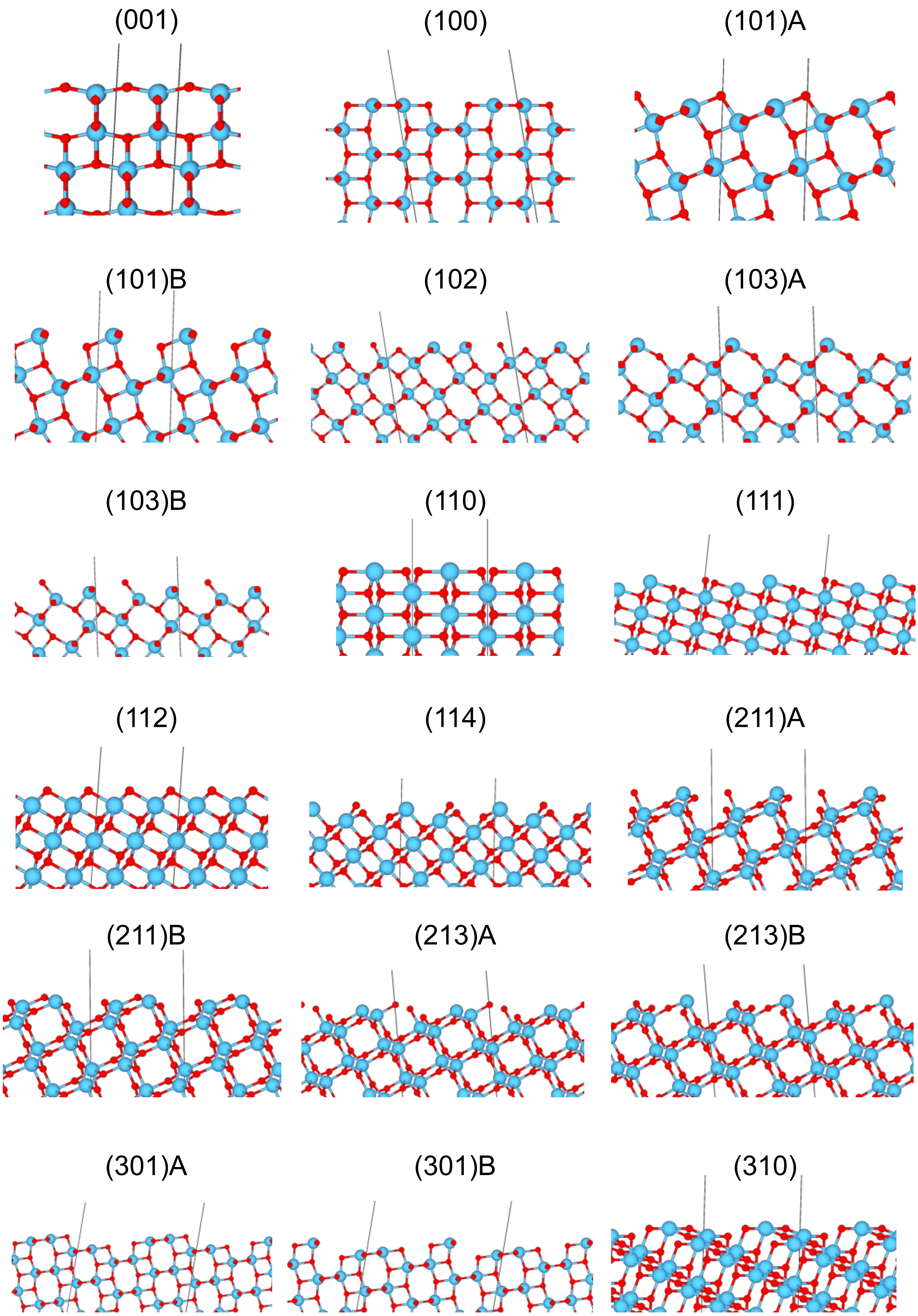

Figure S4. Terminations of cleaved anatase structure TiO_2 slabs. Blue and red balls correspond toTi and O atoms, respectively.

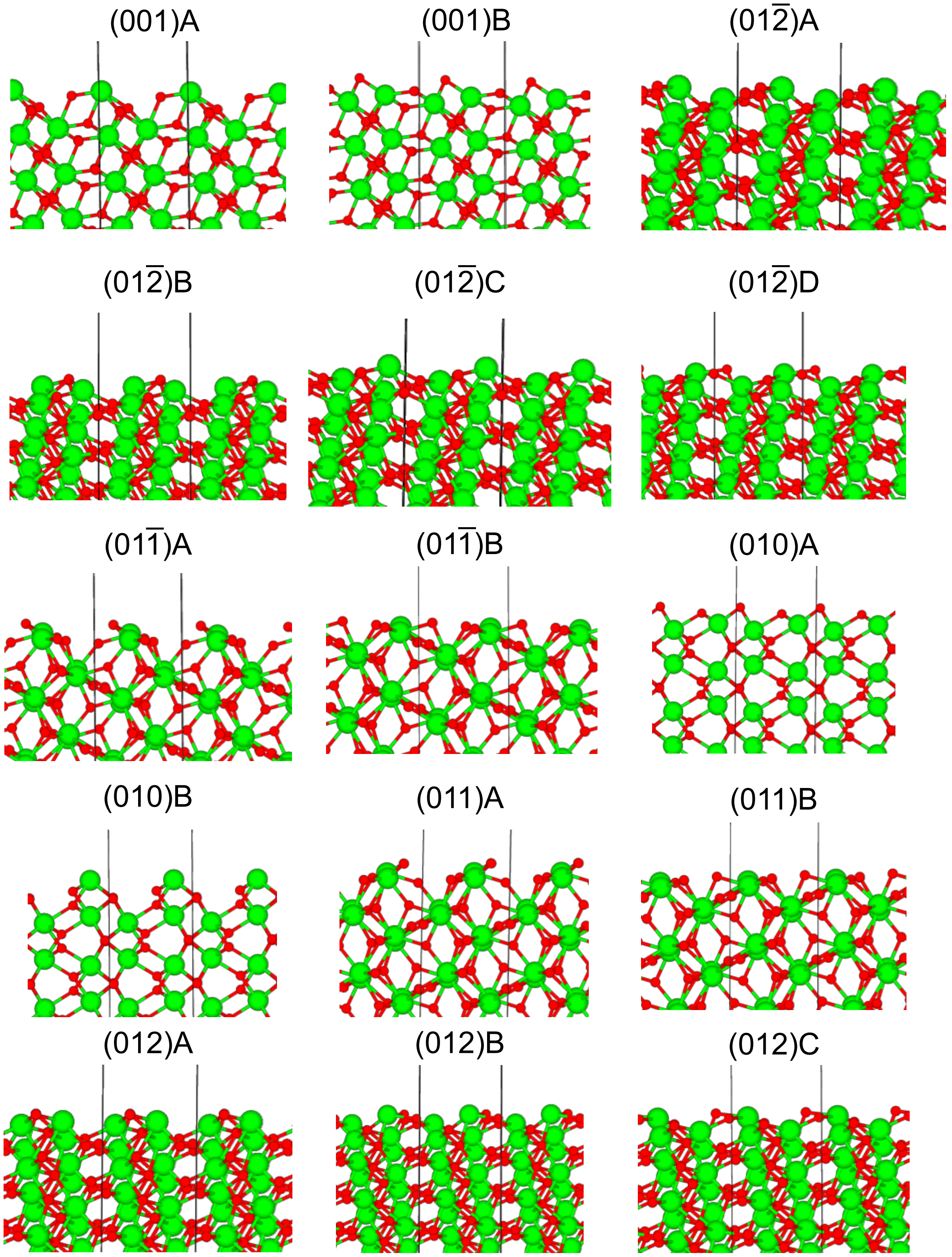

Figure S5. Terminations of cleaved ZrO_2 slabs. Green and red balls correspond to Zr and O atoms, respectively.

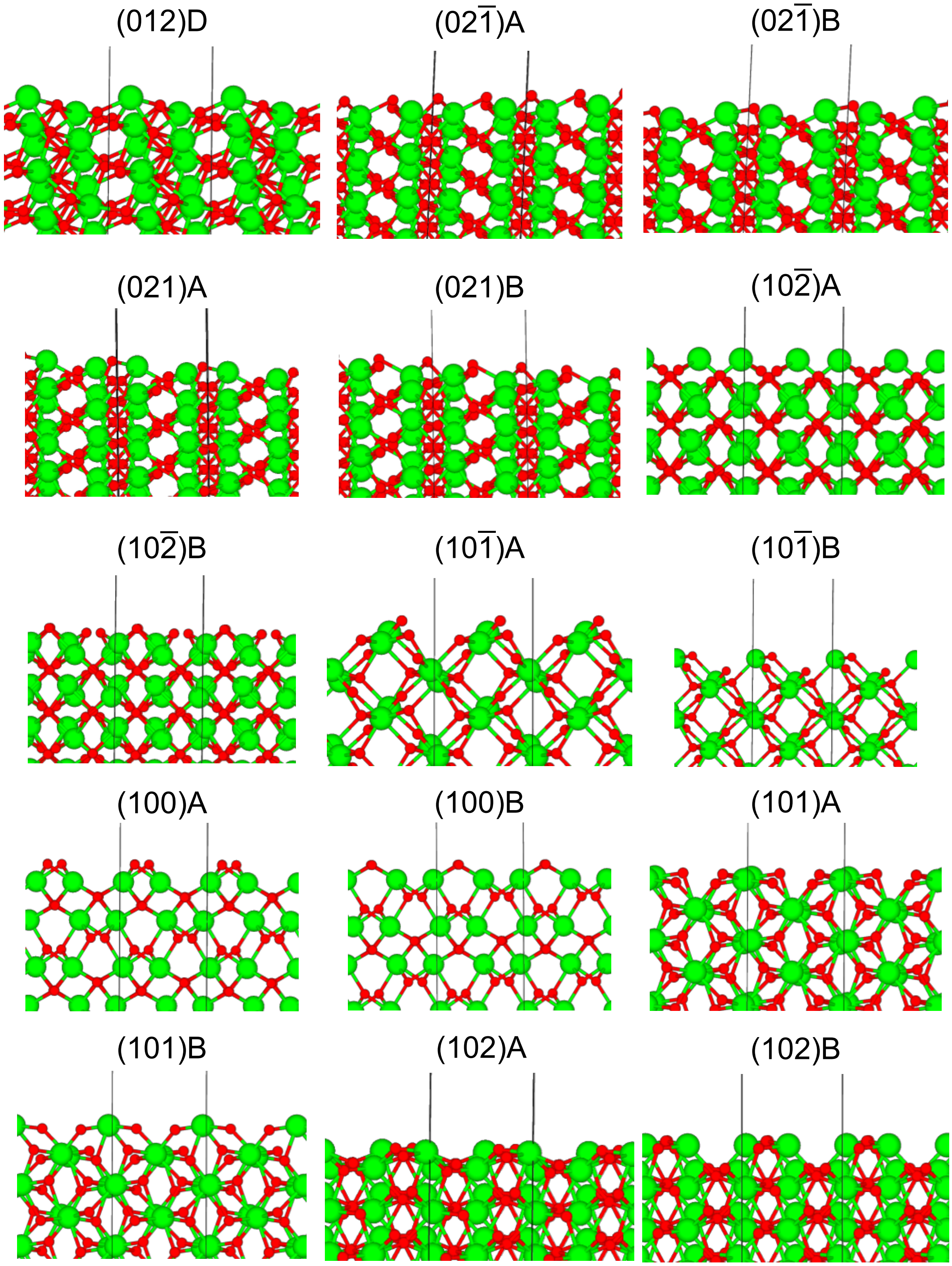

Figure S6. Terminations of cleaved ZrO_2 slabs. Green and red balls correspond to Zr and O atoms, respectively.

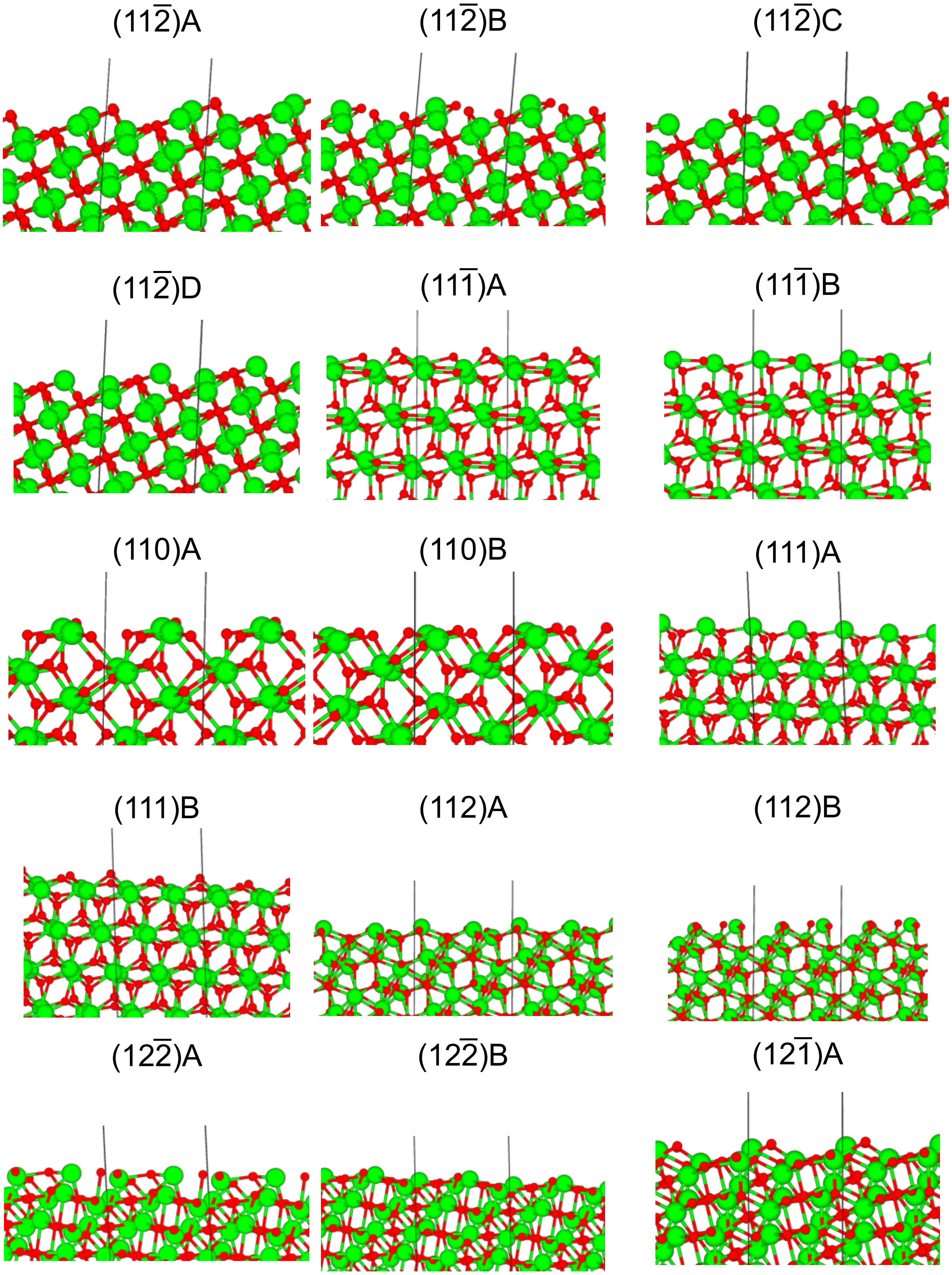

Figure S7. Terminations of cleaved ZrO_2 slabs. Green and red balls correspond to Zr and O atoms, respectively.

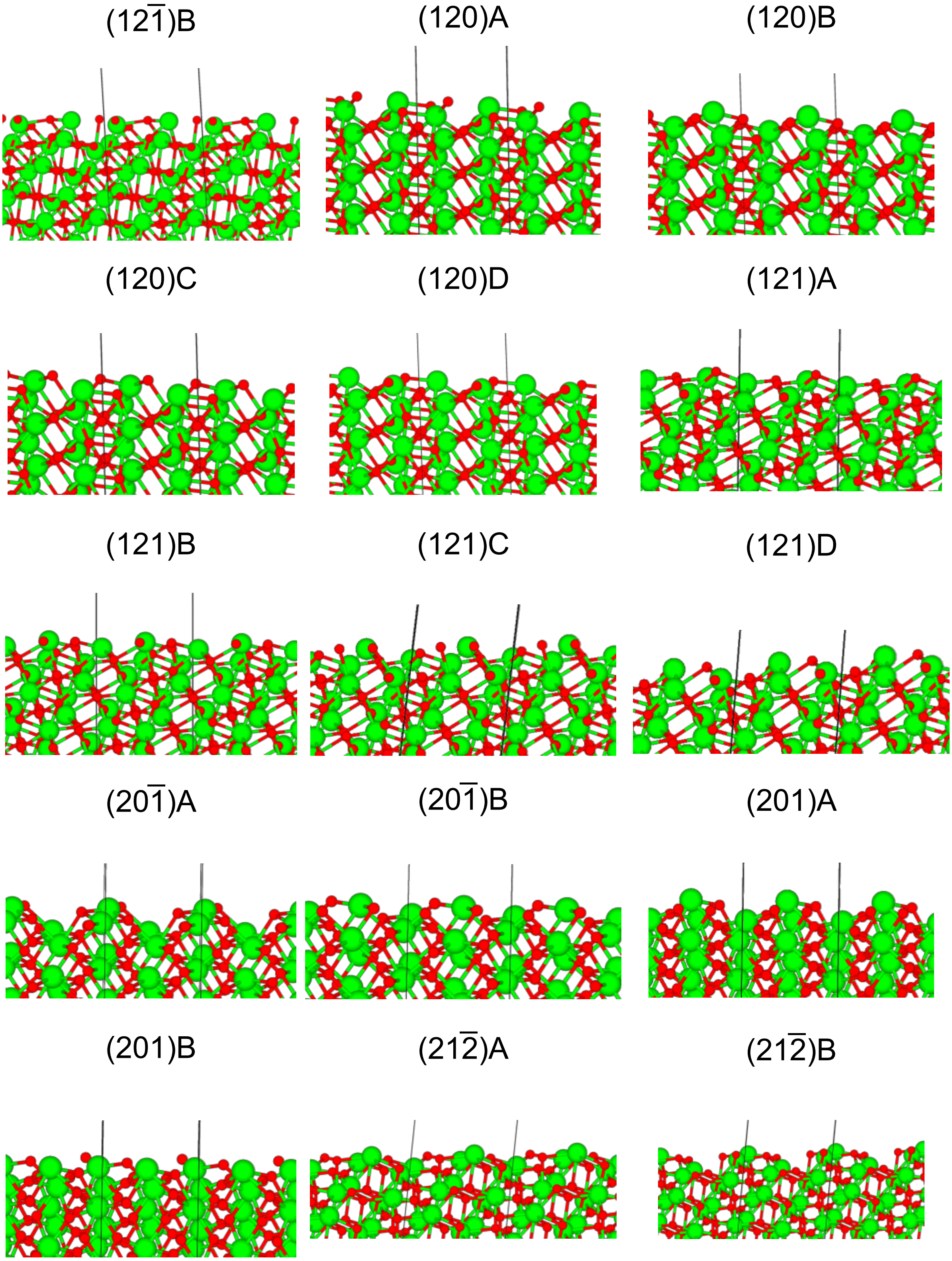

Figure S8. Terminations of cleaved ZrO_2 slabs. Green and red balls correspond to Zr and O atoms, respectively.

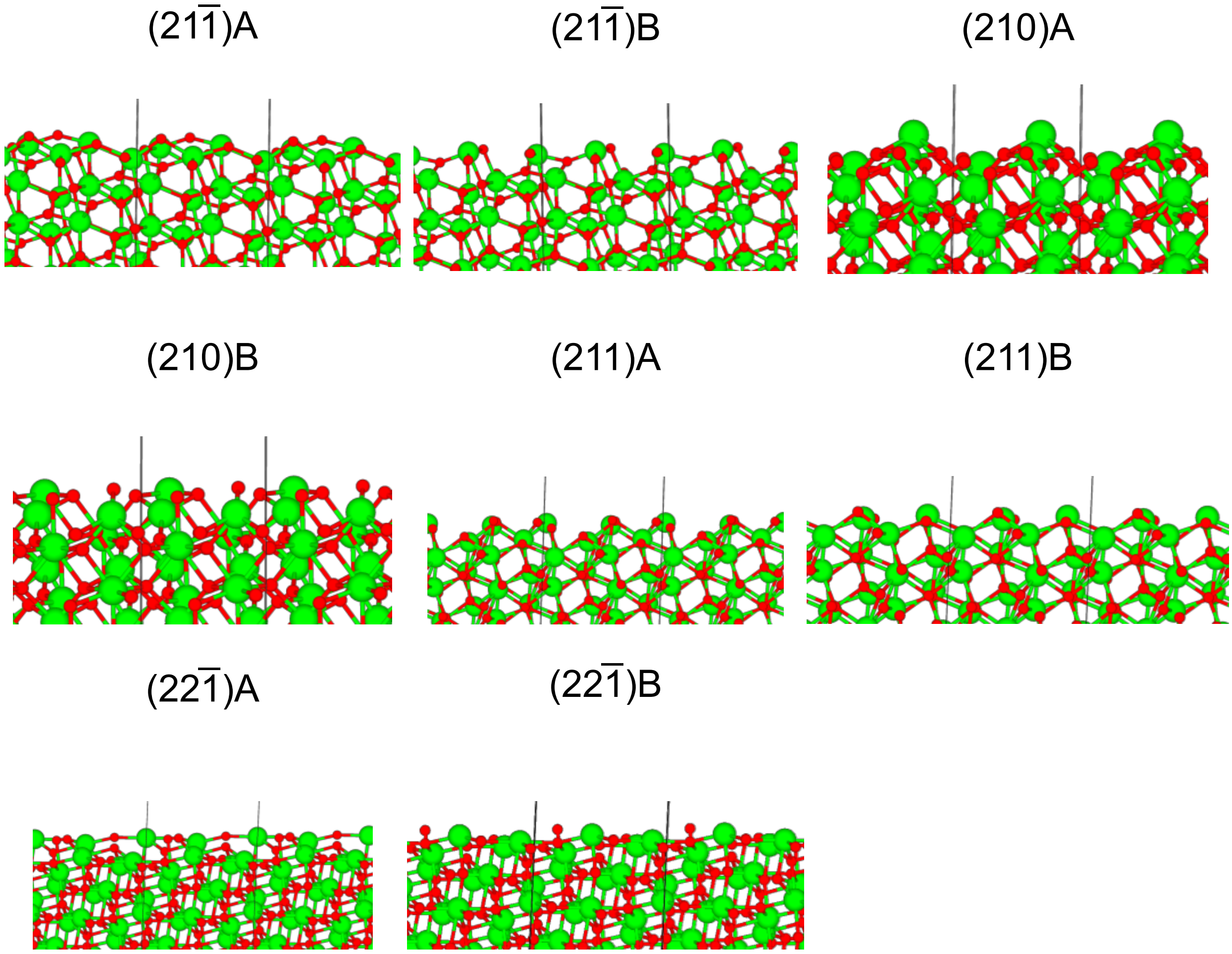

Figure S9. Terminations of cleaved ZrO_2 slabs. Green and red balls correspond to Zr and O atoms, respectively.

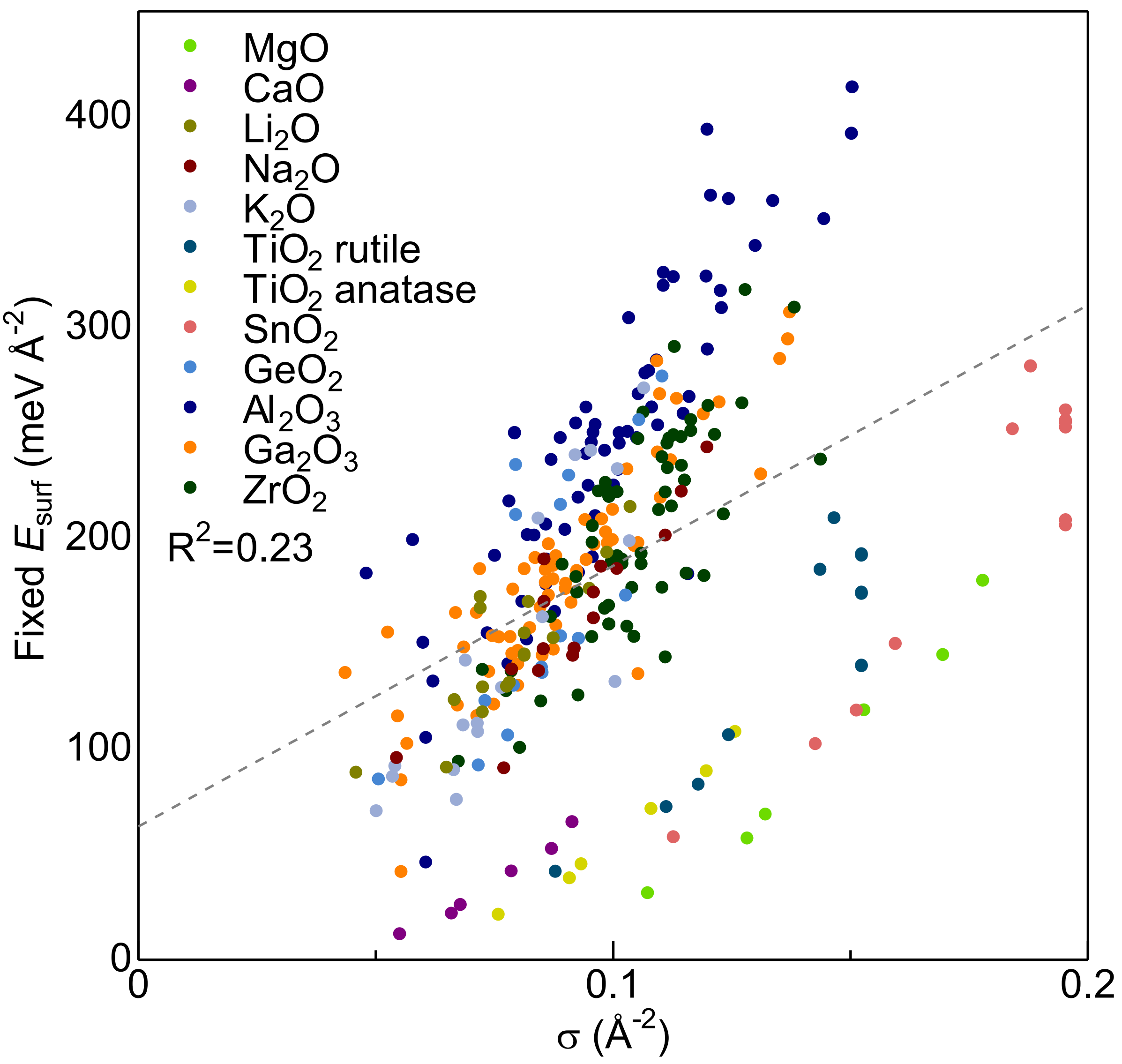

Figure S10. Relation between σ and fixed E_{surf} for all crystals.

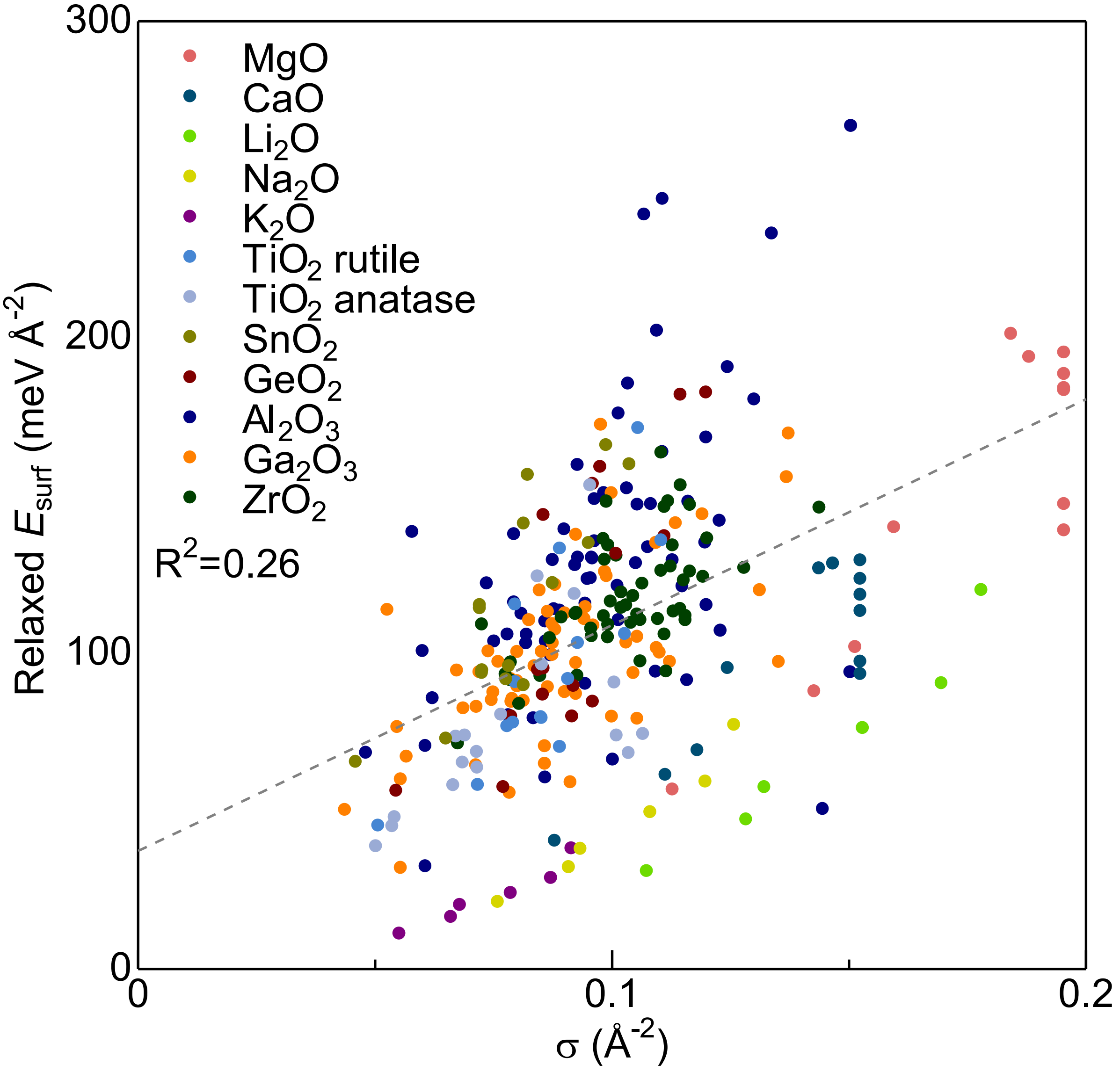

Figure S11. Relation between σ and relaxed E_{surf} for all crystals.
